# Quaternary Habitat Fluctuations and Demographic Dynamics in Turtles Inferred From Environmental Niche Modelling and Whole Genome Data

**DOI:** 10.1111/1755-0998.70040

**Published:** 2025-09-04

**Authors:** Marcella Sozzoni, Jennifer Balacco, Massimo Bellavita, Anna Brüniche‐Olsen, Giulio Formenti, Nivesh Jain, Bonhwang Koo, Jacquelyn Mountcastle, Marc Palmada‐Flores, Vladimir Trifonov, Guido Chelazzi, Sara Fratini, Erich D. Jarvis, Chiara Natali, Davide Nespoli, Claudio Ciofi, Alessio Iannucci

**Affiliations:** ^1^ Department of Biology University of Florence Sesto Fiorentino Italy; ^2^ Vertebrate Genome Laboratory The Rockefeller University New York City New York USA; ^3^ Riserva Naturale Regionale Monte Rufeno Acquapendente Italy; ^4^ Center for Macroecology, Evolution and Climate University of Copenhagen Copenhagen Denmark; ^5^ Laboratory of Neurogenetics of Language The Rockefeller University New York City New York USA; ^6^ Howard Hughes Medical Institute Chevy Chase Maryland USA; ^7^ Tree of Life Wellcome Sanger Institute Cambridge UK; ^8^ Institute of Molecular and Cellular Biology SB RAS Novosibirsk Russia

**Keywords:** effective population size, *Emys orbicularis*, ENM, population genomics, PSMC, reptiles

## Abstract

Quaternary climatic fluctuations had a substantial influence on ecosystems, species distribution, phenology and genetic diversity, driving extinction, adaptation and demographic shifts during glacial periods and postglacial expansions. Integration of genomic data and environmental niche modelling can provide valuable insights on how organisms responded to past environmental variations and contribute to assessing vulnerability and resilience to ongoing climatic challenges. Among vertebrates, turtles are particularly vulnerable to habitat changes because of distinctive life history traits and the effect of environmental conditions on physiology and survival. We estimated contemporary heterozygosity (*H*) and effective population size (*N*_e_) using a high‐quality chromosome‐level reference genome we produced for the European pond turtle ( *Emys orbicularis* ) and reference genomes and whole genome sequence data available for 21 species of tortoises and freshwater turtles. We implemented environmental niche modelling (ENM) to estimate past habitat dynamics. We found recurrent cycles of population expansion and contraction over the last 10 Mya in all species, with a general pattern of decrease in *N*_e_ correlated with temperature reduction after the last interglacial period. No correlation was found between habitat fluctuations during the Quaternary and past *N*_e_. Moreover, neither *H* nor mean *N*_e_ was correlated to threat status as defined by IUCN Red List categories. Our results add to studies on other vertebrates showing the extent to which genetic parameters can aid the assessment of conservation status, and although genomic data may not always be consistent indicators of the level of threat, investigations of which genomic parameters could best represent essential biodiversity variables should be consistently supported.

## 1 Introduction

The recurrent climatic fluctuations of the Quaternary led to a progression of glacial and interglacial periods characterised by repeated changes in environmental conditions and resource availability (Ehlers et al. 2018). As a consequence, several biotopes disappeared entirely or changed considerably, leading to either extinction, survival in glacial refugia or adaptation to new habitats (Hofreiter and Stewart 2009; Seersholm et al. 2020). The global eustatic sea level variations of colder climate phases also resulted in the connection of previously isolated landmasses due to the exposure of continental shelves and a consequent habitat expansion for many organisms (Hofreiter and Stewart 2009; Weigelt et al. 2016).

The environmental and distribution shifts that most species suffered during the Pleistocene also had genetic consequences as a result of bottlenecks in glacial refugia, followed by genetic drift and differentiation among populations or founder effects during postglacial expansion (Hewitt 2004; Niedziałkowska et al. 2016; Polfus et al. 2017; de Lafontaine et al. 2018; Manthey et al. 2021; Wielstra et al. 2021; Kumar et al. 2023; Winker et al. 2023).

Patterns of demographic variation are reflected in the genome of a species, and can be examined to estimate fluctuations in effective population size, *N*_e_ (Li and Durbin 2011; Beichman et al. 2018). A frequently used method is the Pairwise Sequentially Markovian Coalescent (PSMC), which makes use of observed heterozygosity (*H*) and recombination information from a single genome to estimate changes in *N*_e_ over time (Li and Durbin 2011). Moreover, the combination of PSMC and environmental niche modelling (ENM), which allows reconstructing changes in a species' distribution using paleoclimatic data, can provide insights into the effects of environmental variations on the life history of a species and eventually help understand its vulnerability to climate changes (Chattopadhyay et al. 2019; Sang et al. 2022).

Among vertebrates, turtles are one of the most endangered groups, primarily due to the illegal trade and capture for consumption, habitat encroachment and pollution as well as anthropogenic climate change. Vulnerability is further exacerbated as a result of distinctive life history traits including delayed maturity, extended reproductive life and longevity (Stanford et al. 2018, 2020; Rhodin et al. 2021). Current climatic alterations are already affecting a number of physiological and phenological characteristics of this taxon. In marine turtles, higher than usual temperatures are responsible for feminisation and increased nest mortality, while freshwater turtles (terrapins) are experiencing particularly short incubation times and early hatchling emergence (Patrício et al. 2021; Santoro et al. 2023). These factors alter individual survival rates and lead to demographic decline (Miller‐Rushing et al. 2010; Burkhart et al. 2017).

Whole genome sequencing (WGS) studies have so far characterised the complete genomes of approximately 40 species of turtles (NCBI Genome Database 2024), however, no attempts were made to work on an integrated framework based on genomics and environmental modelling to investigate demographic histories of extant populations and try to understand how climate change could eventually affect future generations.

We aimed at investigating how past variations in the extent of suitable habitats affected demographic patterns and genomic diversity in terrapins and land tortoises during the Pleistocene. We produced the first chromosome‐level reference genome (*sensu* Formenti et al. 2022) for the European pond turtle ( *Emys orbicularis* ) and performed a comparison among *E. orbicularis* and 21 other published genomes of terrapins and tortoises. We used WGS data to estimate current genome heterozygosity and infer past *N*_*e*_ through time. We then implemented an ENM to estimate suitable habitats for each species during the past 1 Mya and investigated whether the demographic history of tortoises and terrapins followed patterns driven by climate changes over time (Bofill and Blom 2024). In particular, we expected *N*_*e*_ to decrease during glaciations and increase during interglacial periods. Similarly, demographic trends could mirror ecological niche dynamics in terms of a decrease in *N*_e_ and *H* during times of reduced habitat availability and a relative increase during periods of expansions. Moreover, we explored whether species listed as threatened in the IUCN Red List had a lower contemporary *H* and mean past *N*_*e*_ than non‐threatened species. This pattern was observed in other vertebrate groups whereby *N*_e_, *H* and runs of homozygosity (ROH) were often associated with IUCN Red List categories, suggesting a role of genomic metrics as proxies to evaluate the conservation status of species lacking formal assessments (e.g., Brüniche‐Olsen et al. 2019, 2021; Vitorino et al. 2019; Wilder et al. 2023; Li et al. 2025). Our results are an example of how the integration of genomic signatures of past demographic trends and fluctuations in environmental factors may help elucidate life history traits associated with environmental change and provide a framework to better understand possible adaptation of heterotherm organisms, such as turtles, to future habitat changes.

## 2 Materials and Methods

### 2.1 The Reference Genome of the European Pond Turtle

The European pond turtle, *E. orbicularis* , is a non‐migratory terrapin distributed from the Mediterranean Maghreb basin in northwestern Africa through most of southern, central and eastern Europe to Latvia, Asia Minor and the Caspian and Aral Seas (Khabibullin 2004; Mazanaeva and Orlova 2004; Fritz et al. 2007, 2009; Pupins and Pupina 2008; Spinks and Shaffer 2009; Velo‐Antón et al. 2015; El Hili et al. 2020; Gherbi et al. 2023). Natural populations are found in wetlands, ponds and rivers, as well as artificial water bodies (Zuffi 2000, 2004; Ficetola et al. 2004). Dry land is also important, and female *E. orbicularis* equally depends on loose, pity soil close to aquatic habitats for oviposition (Rovero and Chelazzi 1996; Ficetola and De Bernardi 2006). According to the European Red List of IUCN, the conservation status of *E. orbicularis* is Near Threatened in Europe and Vulnerable within the European Union (Cox and Temple 2009). Population decline is mainly due to habitat loss and encroachment, pollution, competition for biotic and abiotic resources with non‐native terrapins, and pet trade (Berthomieu and Vermeer 2021).

Approximately 200 μL of whole blood was collected using an EDTA coated syringe from a female *E. orbicularis* in central Italy. The blood sample was flash‐frozen in liquid nitrogen and stored at −80°C. High molecular weight DNA was extracted following digestion in a lysis buffer with 200 μg of Proteinase K for 1 h at 55°C. Nucleic acids were bound to magnetic disks covered with a high density of nanostructured silica, placed against a magnetic rack and washed from organic debris and contaminants according to the Nanobind Tissue Big DNA Kit protocol (Circulomics). Whole DNA was then eluted in TE buffer (Tris 10 mM, EDTA 1 mM, pH 8.0) and stored at 4°C. Nucleic acids yield was quantified in a Qubit fluorimeter using a Qubit dsDNA Broad Range assay (Life Technologies) and DNA purity was assessed by comparing absorbance values at 260, 280 and 230 nm in a NanoDrop 2000 spectrophotometer (Thermo Scientific). Integrity of DNA was then assessed by automated pulsed‐field capillary electrophoresis in a Femto Pulse system (Agilent).

Whole DNA was sheared in a Megaruptor 2 DNA shearing system (Diagenode) using large fragment hydropores with a target mean fragment length of 20 kb. Fragments profile was assessed by capillary electrophoresis on a Fragment Analyser using an HS large fragment 50 kb kit (Agilent Technologies). Repair and A‐tailing of DNA fragments, SMRTbell adapters ligation and AMPure PB beads size selection to remove fragments shorter than 5 kb were performed according to the SMRTbell prep kit 3.0 protocol (Pacific Biosciences). Primer annealing, polymerase binding and preparation of internal DNA control were performed using the Pacific Biosciences Sequel II binding kit and DNA internal control complex 3.2 according to the manufacturer's protocol. Sequencing runs were set up using SMRT Link v11.1. Insert size varied from 10 to 15 kb. Samples were sequenced in HiFi mode on a Pacific Biosciences Sequel IIe platform using Sequel II Sequencing plates 2.0 and 8 M ZMW SMRT cells with a 30‐h movie time and 2 h of pre‐extension time for a target 30× genome coverage.

Genome‐wide chromosome structural features were assessed by generating proximally ligated DNA libraries using the Arima HiC+ for Genome‐Wide HiC Kit in vivo cross‐linking with 2‐enzyme proximity ligation. Proximally ligated DNA was sheared in a Covaris M220 focused ultrasonicator, size selected to 200–600 bp using solid‐phase reversible immobilisation (SPRI) paramagnetic beads, labelled with biotinylated nucleotides and enriched using streptavidin‐coated magnetic beads. Sequencing libraries compatible with Illumina sequencing by synthesis technology were generated using the KAPA Hyper Prep kit (Roche). Libraries were amplified through PCR, purified with SPRI beads and sequenced 2 × 150 paired end on an Illumina HiSeq X in order to attain a 60× mean genome coverage. Finally, DNA was labelled for Bionano Genomics optical mapping using the Bionano Prep Direct Label and Stain Protocol with Direct Labeling Enzyme 1 and run on a Saphyr instrument chip flowcell.

Genome assembly was performed using the Galaxy Europe online platform for genomic data analysis (The Galaxy Community 2022) following the Vertebrate Genomes Project assembly pipeline 2.0 (Larivière et al. 2024). Genome size, heterozygosity and repeat content were estimated using statistical analysis of k‐mers profiles in unassembled data (Vurture et al. 2017; Ranallo‐Benavidez et al. 2020) implemented in Genomescope2 (Galaxy Version 2.0 + galaxy1). A 21‐mer profile was generated with Meryl (Galaxy Version 1.3 + galaxy4) using PacBio HiFi reads (Vurture et al. 2017; Ranallo‐Benavidez et al. 2020). Adaptors were trimmed with Cutadapt (Galaxy Version 3.5 + galaxy1) (Martin 2011) and reads were assembled into contigs along with Hi‐C reads using Hifiasm (Galaxy Version 0.16.1 + galaxy2) (Cheng et al. 2022). Two intermediate phased haplotypes were produced, resulting in a complete (primary) assembly with long stretches of phased contigs and an incomplete (alternate) assembly consisting of single contigs of the alternate haplotype in heterozygous regions. BUSCO (Galaxy Version 5.2.2 + galaxy2) (Simao et al. 2015; Manni et al. 2021) was run to assess the presence of vertebrate orthologous genes in both contigged haplotypes. Both assemblies were then scaffolded with Bionano optical maps (Shelton et al. 2015) using the Bionano Solve tool implemented in Bionano Hybrid Scaffolds (Galaxy Version 3.7.0 + galaxy0). Two partially scaffolded assemblies were obtained for both haplotypes. Finally, Hi‐C reads were aligned to the partial assemblies to obtain the final scaffolded assemblies using SALSA (Galaxy version 2.3 + galaxy2) (Ghurye et al. 2017, 2019). Manual curation to resolve misplaced scaffolds was mostly performed using PretextView (Harry 2022) with additional insights provided by HiGlass (Kerpedjiev et al. 2018). Statistics and quality scores were calculated using gfastats (1.2.2 + galaxy0) (Formenti 2022) for each step of the assembly. We also computed the quality metrics proposed by the Vertebrate Genomes Project (Rhie et al. 2021) as x.y.Q.C, where *x* = log_10_[contig NG50], *y* = log_10_[scaffold NG50], *Q* = Quality Value of base accuracy, and *C* = percentage of the assembly assigned to chromosomes. The reference genome was then annotated by NCBI using the eukaryotic genome annotation pipeline (Goldfarb et al. 2025) and publicly available RNA‐seq of *E. orbicularis* (Romiguier et al. 2014).

The reference genome of *E. orbicularis* was partially assigned to the species karyotype using a ChromSeq approach based on physical chromosome isolation and sequencing (Iannucci, Benazzo, et al. 2021; Iannucci, Makunin, et al. 2021). Metaphase spreads with good chromosome definition were obtained from fibroblast cell cultures as described in Iannucci et al. (2019). Slides with metaphase chromosome preparations were stained with a Giemsa solution and mounted on a Zeiss Axio Observer 7 inverted microscope equipped with a rotating table and a right‐handed micromanipulator (Narishige). Chromosomes were manually micro‐dissected using glass needles and inserted into pasteur pipettes filled with a collection drop of proteinase K and a saline solution. Needles and pipettes for micro‐dissection were prepared using a Narishige PC‐10 pipette puller. The tip of each pipette was then broken into a sterile tube and the DNA of each chromosome was amplified using a GenomePlex Whole Genome Amplification Kit (Sigma‐Aldrich). Amplicons were then sequenced paired‐end on an Illumina NovaSeq 6000 system using a NovaSeq 6000 SP Reagent Kit v1.5 (300 cycles). Illumina reads were checked using FastQC (Galaxy Version 0.73 + galaxy0) (Andrews 2010), trimmed of adaptors with Cutadapt and mapped to the *E. orbicularis* reference genome using BWA‐MEM (Galaxy Version 0.7.17.2) (Li and Durbin 2009; Li 2013). The mapped alignments were then filtered for mapping quality with Samtools 1.11 (Danecek et al. 2021) using the default pipeline parameters of the dopseq pipeline (Makunin et al. 2016). Scaffolds of the reference genome were then assigned to specific chromosomes of the *E. orbicularis* karyotype based on a minimum number of positions where chromosome reads were mapped to the scaffold and the distance between these positions along the scaffold. Several statistics were calculated for each scaffold (Table S1). These included mean pairwise distance between positions on scaffold, mean number of reads per position on scaffold, number of positions on scaffold, position representation ratio (PRR) and *p* value of PRR. The PRR of each scaffold was used to evaluate enrichment of a given scaffold on chromosomes and was calculated as the ratio of positions on scaffold to positions in genome divided by the ratio of scaffold length to genome length. A PRR > 1 corresponded to enrichment, while PRR < 1 was equal to depletion. As the PRR value is distributed lognormally, we used its logarithmic form in our calculations. The *p* value of PRR was estimated using a binomial test. To filter out statistically significant PRR values only, we used a threshold of logPRR > 0 and its *p* value ≤ 0.01. Scaffolds with logPRR > 0 were considered enriched in the given sample (Lind et al. 2019).

### 2.2 Collection of Genomic, Life History and Ecological Data of Terrapins and Tortoises

Genomic data for 21 terrapins and tortoises were downloaded from NCBI (Table S2). For each species, we retrieved short and long reads and genome assembly (Table S2). We used FastQC to generate summary statistics and check for the presence of adapters, which were trimmed using Cutadapt. Short reads were aligned to the reference genome assemblies using BWA‐MEM. Long reads were aligned using the map‐hifi option implemented in Minimap2 (Galaxy Version 2.24 + galaxy0) (Li and Durbin 2010). For each species, we quantified the genome‐wide heterozygosity, that is the proportion of heterozygote genotypes divided by the genome size based on the folded site frequency spectrum (sfs), using angsd v0.940 (Korneliussen et al. 2014). We used filters on the base quality score (−minQ 20) and minimum mapping quality (−minMapQ 30).

We then compiled a life history table including information on age at sexual maturity, reproductive longevity, generation time, conservation status, habitat and climatic zone (Table S2). Data on age at maturity and average reproductive longevity were obtained from the Amniote Database (Myhrvold et al. 2015), the IUCN Red List of Threatened Species (IUCN 2022) and from the literature (see Table S2). We calculated an average age at sexual maturity when a range of different values was reported for a certain species. Generation time (g) was calculated as the age of maturity plus half the reproductive longevity (Pianka 1994; Fitak and Johnsen 2018). Conservation status for each species was obtained from the IUCN Red List of Threatened Species or the Tortoise and Freshwater Turtle IUCN Specialist Group report (Rhodin et al. 2021; IUCN 2022). Information on habitat type for each species was retrieved from the literature (Table S2) and climatic zone data were obtained from an updated version of the Köppen–Geiger climate classification (Kottek et al. 2006) (Table S2).

### 2.3 Demographic Analysis

We estimated *N*_e_ trajectories through time using PSMC v0.6.5‐r67 (Li and Durbin 2011). The software models coalescent events in time between two haplotypes of a genome in order to infer demographic history. We first used Samtools 1.11 to infer the mean depth of coverage (DOC) of the .bam files. Nucleotide sites with less than one‐third and more than double the mean DOC were filtered out. Bcftools 1.9 (Danecek et al. 2021) was used to call single nucleotide polymorphisms, and the vcfutils.pl tool implemented in Samtools 1.11 was used to convert the variant call format into masked fasta format (.psmcfa file). The effective population size was inferred over 64 atomic time intervals distributed across 28 free intervals. The first four atomic intervals were grouped in two free intervals. Fifty atomic intervals were grouped in pairs in 25 free intervals, while an additional four and six atomic intervals were grouped each in one free interval. PSMC parameter values were set to −p = ‘2 + 2 + 25*2 + 4 + 6’, −*t*15 and −*r*5, where −*t* corresponds to the upper temporal limit for the most recent common ancestor and −*r* is the ratio between genomic diversity (*θ*) and the recombination rate (*ρ*) (Fitak and Johnsen 2018; Vilaca et al. 2021; Liu et al. 2022; Ren et al. 2022). Variance of *N*_*e*_ estimates was inferred after 30 bootstrap replicates.

To convert PSMC trajectories from generations into years, we calculated, for each species, the pairwise genomic distance between that species and the most recent ancestor according to the divergence time used in the phylogenetic reconstruction of Thomson et al. (2021). We used angsd v0.940 with consensus base call (–doIbs 2) and applied filters on the base quality score (−minQ 20) and minimum mapping quality (−minMapQ 30). We also removed bad reads (−remove_bads 1) and printed a distance matrix (−makematrix 1). If the most recent node comprised multiple species, we evaluated the distance between each pair of species and then calculated the mean value. Genomic distances were then scaled to divergence time by calculating the genome‐wide mutation rate as *μ* = (pairwise genomic distance × generation time)/(2 × divergence time) (Nei 1987).

Patterns of overall demographic fluctuations were investigated by using *N*_*e*_ values of the demographic trajectories ranging from 10 Mya to 10 kya. We used a min‐max normalisation to rescale values between 0 and 1. We plotted the overall mean *N*_*e*_ trajectory and the mean *N*_*e*_ trajectory of species grouped by habitat (freshwater or terrestrial) and climatic zone (tropical or temperate) to assess differences between categories. In each plot, we included data on mean surface temperature taken from Hansen et al. (2013). Plots were generated using ggplot2 v 3.4.4 (Wicham 2016) in Rstudio 2022.12.0 build 353 (R version 4.2.2) (RStudio Team 2020; R Core Team 2022) following Brüniche‐Olsen et al. (2021).

### 2.4 Environmental Niche Modelling

Global occurrence of each species was retrieved from the Global Biodiversity Information Facility (GBIF 2023) using the function occ_search of the R package ‘rgbif’ v3.7.5 (Chamberlain et al. 2023). For each species, we downloaded a maximum of 3000 occurrence records based on field observations and geo‐localised information. A species was excluded if less than 15 occurrences were available in the database. Macro‐climatic data were obtained for 19 bioclimatic variables (Bioclim1‐19) from the WorldClim database v2.1 (Hijmans et al. 2005) at a spatial resolution of 2.5 arc minutes. Occurrences were then thinned using the R package spThin (Aiello‐Lammens et al. 2015) to a minimum distance of 4.5 km, matching the raster resolution of the bioclimatic variables. For each species, climatic raster maps were reduced to occurrence locations with a 10° latitude and longitude buffer. Since bioclimatic variables have a high level of collinearity, a principal component analysis (PCA) was performed in order to reduce dimensionality and correlation among predictors using the rasterPCA tool of the R package RSToolboox v0.3.0 (Bonet et al. 2019). For each species, we selected the first six principal components that explained more than 90% of variation.

Environmental niche models of different complexity were compared using a Maxent algorithm based on the automated calibration and evaluation protocol implemented in the ENMeval R package 2.0.4 (Kass et al. 2021). This method allows for independent training and testing data subsets to reduce data overfitting due to sampling bias (Muscarella et al. 2014). We fitted models using all nine combinations of linear, quadratic and product feature classes (Brüniche‐Olsen et al. 2021; Merow et al. 2013). We excluded feature classes Hinge and Threshold for they required information on physiological tolerance (e.g., thermal limits) that were not available for our species (Merow et al. 2013). We then used three possible regularisation multipliers (1, 2 and 5) in order to fit models including the minimum, mean and maximum values of the regularisation multipliers available (1 to 5).

We then used 10,000 random background points for each model, which was subsequently evaluated using a random *k*‐fold approach with *k* = 4. The random *k*‐fold method splits the data into *k* independent, equal‐size subsets. For each subset, the model is trained with *k* − 1 subsets and evaluated on the *k*‐th subset (Merow et al. 2013). The best model for each species was selected by using the area under the curve (AUC) of the receiving operating characteristics and the Akaike information criterion (AIC). Values of AUC range from 0 to 1, with larger values representing more accurate predictions. An AUC value of 0.5 indicates that the model prediction is no better than random; values between 0.6 and 0.7 imply poor model performance, values between 0.7 and 0.9 indicate moderate performance, while values above 0.9 express high model performance (Araújo et al. 2005). We selected models with the lowest AIC (calculated for the training dataset) and AUC higher than 0.7 (calculated for the test dataset) (Burnham and Anderson 2004; Elith et al. 2006). We produced habitat prediction maps using top fitted models and the function enm.maxnet@predict from the ENMeval package for present and four past climate scenarios, including Early Holocene, the Last Glacial Maximum (LGM), Last Interglacial (LIG) and Marine Isotope Stage 19 Interglaciation (MIS19), considered the closest analogue to the current interglacial (Brown et al. 2018). For MIS19, we excluded variables BIO2, BIO3, BIO5, BIO6 and BIO7, which could not be retrieved. The raster maps were cropped in order to exclude regions covered by ice. Maps of species distribution were produced by calculating the suitable area available as the area of all raster cells with medium to high probability (i.e., 0.36–1.00) to find a species (Chattopadhyay et al. 2019; Brüniche‐Olsen et al. 2021). We were not able to produce an ENM for three species with little occurrence data available ( *Chelonoidis abingdonii* , *Cuora mccordi* and *Rafetus swinhoei* ). We finally checked for the presence of residual spatial autocorrelation using Moran's *I* calculation at 21 distance points between 0 and 10 km. There was no significant spatial autocorrelation under the 4.5 km threshold (data not shown). All analyses were performed using Rstudio 2022.12.0 build 353 (R version 4.2.2) following Brüniche‐Olsen et al. (2021).

### 2.5 Correlation Between ENM and PSMC

Correspondence between temporal fluctuations in *N*_*e*_ and occurrence based on past climatic conditions was investigated across two time periods spanning from MIS19 (~787 kya) to LIG (130 kya) and from LIG to LGM (20 kya), respectively, for which information on both *N*_*e*_ and distribution was available. The selected time periods span a total of approximately 760 kya and fall beyond the most recent 300 to 400 generations for which the PSMC estimates are less reliable (Nadachowska‐Brzyska et al. 2022). For each period, we calculated changes in *N*_*e*_ and area availability between the two extremes by subtracting the *N*_e_ and suitable habitat of the most recent period from the values of the previous period. Effective population size and area availability were then coded as increasing, decreasing, or stable if resulting values were negative, positive, or equal to 0, respectively. We then created a contingency table (Table S3), which was used to check for association between the extent of suitable habitat and *N*_*e*_ using a chi‐squared independence test. We calculated the Phi coefficient of correlation to determine the strength of association between ENM and PSMC results. Values of Phi vary between −1, denoting a negative association, and +1 suggesting a strong positive association. Considering that the Phi test requires binomial data, we excluded three species ( *Emys orbicularis* , *Terrapene carolina triunguis* and *Gopherus evgoodei* ) for which three codes (increasing, decreasing and stable), instead of two, were reported for either *N*_*e*_ or area availability. The analysis was also conducted by pooling species by habitat (aquatic or terrestrial) and climatic zone (tropical or temperate).

We also performed a chi‐squared independence test to check for a relationship between average global temperature changes from MIS19 to LGM and *N*_*e*_ for all the species for which we produced an ENM (Table S4). The analysis was again performed by grouping species by either habitat type (aquatic or terrestrial) or climatic zone (tropical or temperate).

The demographic trajectories, environmental niche and amount of available area for each species were merged using ggplot2 v3.4.4 in Rstudio 2022.12.0 build 353 (R version 4.2.2) (RStudio Team 2020; R Core Team 2022) following Brüniche‐Olsen et al. (2021). In each plot, we included information on mean surface temperature obtained from Hansen et al. (2013).

### 2.6 Multiple Regression Modelling

We investigated the relationship between *H* and mean *N*_e_ (response variables), conservation status and habitat suitability (predictors variables) through three different multiple regression models fitted using phylogenetic generalised least squares (PGLS) regressions. Phylogenetic relationships between species were retrieved from Thomson et al. (2021). Mean *N*_e_ was calculated from the PSMC trajectories for each species over the entire inferred time period (approximately 1 Mya). Very recent PSMC estimates of *N*_e_ tend to be noisy due to a relatively lower number of coalescent events (Li and Durbin 2011; Nadachowska‐Brzyska et al. 2022). For each species, we therefore excluded the four most recent time points of *N*_e_ from the estimation of the mean values (Leroy et al. 2021).

In the first model, we used the current available area to model recent heterozygosity. Similarly, we used the mean available area over the four time periods to model the mean past *N*_e_. For the first class of models conservation status was categorised as ‘Not at risk’ (species listed as Least Concern and Near Threatened) or ‘At risk’ (species listed as Vulnerable, Endangered and Critically Endangered). We then tested the differences in *H* and mean *N*_e_ between different conservation status categories using a Tukey honestly significant difference test and plotted the modelled relationships between *H* and current suitable area and between mean *N*_e_ and past mean area availability using ggplot2 v3.4.4. For the second multiple regression model, we focused on species at risk only. All other variables were as in the first model. The third model class was built on conservation status and suitable habitat. All analyses were performed using the function gls of the package nlme v3.1.161 in Rstudio 2022.12.0 build 353 (R version 4.2.2) following Brüniche‐Olsen et al. (2021).

## 3 Results

### 3.1 A Chromosome‐Level Reference Genome of *Emys orbicularis*

We generated the first chromosome‐level reference genome and an alternate assembly for the European pond turtle using a total of 30‐fold coverage in single‐molecule HiFi long reads, 109‐fold coverage in chromosome conformation Hi‐C, and 229‐fold coverage in optical whole genome map (raw data and assembly accession identifiers are reported in Table 1). Manual assembly curation corrected 46 missing/mis‐joints (making 40 missing joints and breaking 6 mis‐joints), removed 16 haplotypic duplications, reduced the assembly size by 0.03%, and the scaffold number by 33.3%, and increased the scaffold N50 by 6.5%. We assembled 99.7% of sequences in 25 chromosome‐level scaffolds equivalent to 2,303,373,627 base pairs (Table S5). The number of autosomes matched the karyotype of *E. orbicularis* (2*n* = 50), which lacks sex chromosomes, reconstructed by Iannucci et al. (2019). No contaminant sequence was found in the genome assembly. The size of the final reference assembly (rEmyOrb1.pri) was 2.32 Gb and contained 106 scaffolds, with a scaffold N50 of 146 Mb, N90 of 34 Mb and a GC content of 44.70% (Table 1). The assembly had a BUSCO completeness of 99.3% (single 98.1%, duplicated 1.2%) using the sauropsida_odb10 reference set (*n* = 7480). Our assembly reached the quality metrics of 8.8.Q65.C99, which were higher than the quality standards proposed by the Vertebrate Genomes Project (6.7.Q40.C90) (Figures S1–S4). Using a ChromSeq approach, we successfully physically isolated and sequenced six *E. orbicularis* chromosomes, which were assigned to six scaffolds of the genome for a total of 1.30 Gb (55% of total 2.32 Gb assembly) (Tables S1, S6 and S7; Figure S4). The mean coverage for each assigned scaffold was between 2 and 17 (Table S6).

**TABLE 1:** Genome data and assembly statistics for *Emys orbicularis* , rEmyOrb1.hap1.

Project accession data	
Assembly identifier	rEmyOrb1.hap1
Species	*Emys orbicularis*
Specimen	rEmyOrb1
NCBI taxonomy ID	82168
BioProject	PRJNA919152
BioSample ID	SAMN31805221
Isolate information	Female, whole blood
Raw data accession	
Pacific Biosciences SEQUEL IIe	SRR25743592–SRR25743606
Bionano DLS	https://www.genomeark.org/vgp‐all/Emys_orbicularis.html
Arima Hi‐C v2	SRR25743607
Genome assembly	
Assembly accession	GCA_028017835.1
Accession of alternate haplotype	GCA_028017845.1
Assembly size	2.32 Gb (2,324,111,602 bp)
Number of contigs	254
Contigs N50	91.3 Mb
Number of scaffolds	106
Scaffolds N50	146 Mb
Scaffolds N90	34 Mb
Longest scaffold	373 Mb
GC content	44.70%
BUSCO^a^ scores	C: 99.3% [S: 98.1%, D: 1.2%], F: 0.1%, M: 0.6%, *n*: 7480

^a^ BUSCO scores based on the sauropsida_odb10 set using Galaxy Version 5.2.2. C, complete [S, single copy; D, duplicated]; F, fragmented; M, missing; *n*, number of orthologues in comparison.

Annotation using the NCBI's EGAP predicted a total of 22,788 genes and pseudogenes (19,783 protein‐coding and 1794 noncoding). In total, 21,594 transcripts were annotated. A full genome annotation report is available at the NCBI website (https://www.ncbi.nlm.nih.gov/refseq/annotation_euk/Emys_orbicularis/GCF_028017835.1‐RS_2024_05/). The genome and resulting annotations from this project currently represent the NCBI RefSeq genome and annotations for *E. orbicularis* .

### 3.2 Effective Population Size Fluctuations

**FIGURE 1:**
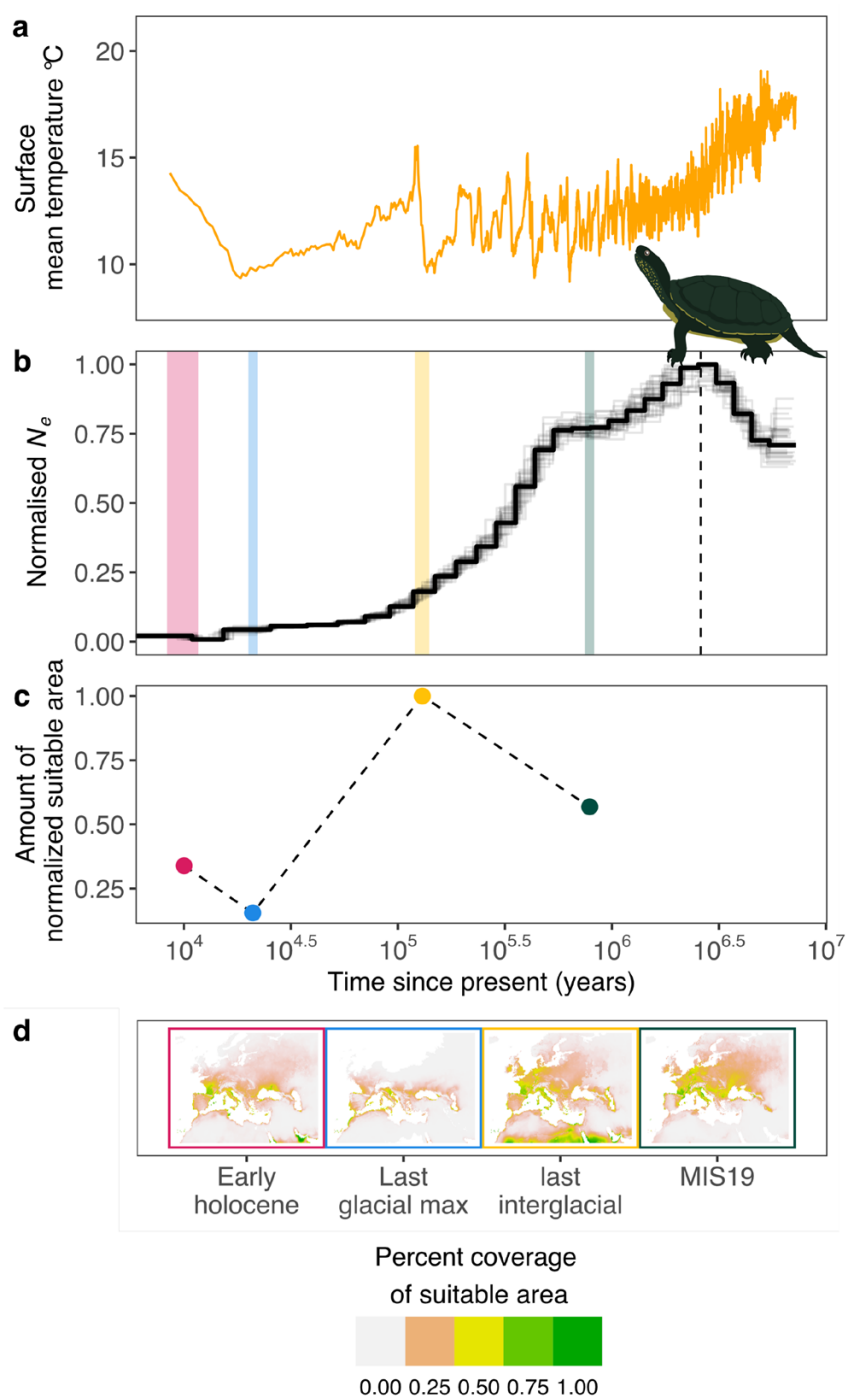
Example of effective population size (*N*_*e*_) trajectories over time and Environmental Niche Modelling (ENM) for the European pond turtle *E. orbicularis*. The orange line in (a) represents the mean surface temperature estimated for the last 10 Mya. A pairwise sequentially Markovian coalescent (PSMC) was used in (b) to estimate *N*_e_ variation over the past 10 Mya. The black line shows *N*_e_ values over time and light grey lines represent PSMC bootstrap replicates. Coloured vertical bars correspond, from left to right, to the Early Holocene, Last Glacial Maximum, Last Interglacial and Marine Isotope Stage 19 Interglaciation (MIS19). The dashed line represents the first appearance of a permanent Antarctic ice cap. Extent (c) and percent coverage (d) of suitable area estimated using ENM are reported from 10 Kya to 1 Mya. Values of *N*_e_ and suitable area were normalised by dividing all values by the maximum value estimated over the four time periods.

**FIGURE 2:**
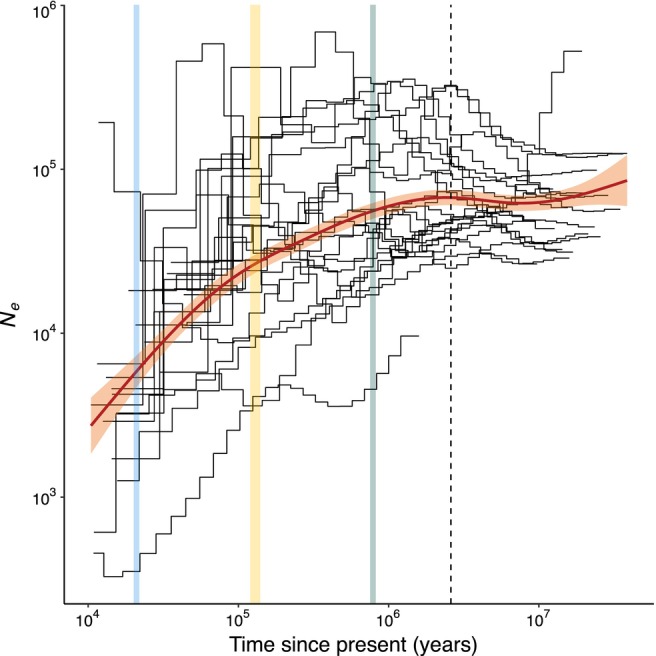
Mean change in *N*_*e*_ from 10 kya to 10 Mya for 22 species of terrapins and tortoises. Each black step line shows changes in *N*_*e*_ through time for a single species. The red line represents the regression model showing the general pattern of *N*_*e*_ variation through time. The light red‐shaded area represents the 95% confidence intervals of the linear model prediction. Coloured vertical bars and dashed vertical line as in Figure 1.

We reconstructed the demographic history of 22 species of terrapins and tortoises from 10 thousand to 10 million years ago. Per year mutation rate ranged from 2.62 × 10^−10^ to 7.83 × 10^−10^ per nucleotide site (Table S8). The per generation mutation rate used for the PSMC spanned from 4.54 × 10^−10^ to 1.14 × 10^−8^ per site (Table S9). Demographic trajectories showed that all species experienced a highly variable *N*_*e*_ at least once from 10 Mya to 100 kya. There was considerable variability of *N*_*e*_ over time among species. Minimum values ranged from *N*_*e*_ = 327 for *Chelonoidis abingdonii* (now extinct, originally from the Pinta Island of the Galápagos archipelago) to *N*_*e*_ = 122,000 for *Pelusios castaneus* (Figure 1; Figures S5–S25). Similarly, maximum *N*_*e*_ estimated over time spanned from *N*_*e*_ = 9600 for *C. abingdonii* (Figure S23) to *N*_*e*_ = 688,000 for *Platysternon megacephalum* (Figure S19). On a global scale, mean normalised *N*_*e*_ for all 22 species showed a slow decrease after the end of the MIS19 and then sharply declined after the end of the LIG (Figure 2). In tropical and temperate regions, we recorded a slight difference in average *N*_*e*_ fluctuations, with temperate species showing a peak in *N*_*e*_ before the MIS19 that we did not see in tropical species. There was also a different pattern between terrestrial and freshwater habitats, with terrestrial species experiencing a period of stability in *N*_*e*_ right before a drastic decrease at the end of the LIG (Figure S26).

### 3.3 Ecological Niche Modelling Variation

A total of 18 MaxEnt models (9 generated for the general model and 9 for the MIS19 only) were built with different parameter values of regularisation multiplier values and feature classes for each of the 19 species for which occurrence data was available. Among the models retained after the validation process, all but two had high AUC scores (> 0.80; Table S10), indicating good discrimination power (Burnham and Anderson 2004; Elith et al. 2006).

The six most important predictors retained after the PCA showed different values across species (Tables S11 and S12). The first principal component of the model constructed using present time data had the highest loading linked to temperature variables for 89% of the species. The most recurrent variable (58%) was the minimum temperature of the coldest month (BIO‐6, Table S11). On the other hand, the variable with the highest loading for the first component of the second model constructed on the MIS19 differed among species. The most recurrent one was the annual precipitation found in 63% of the species (BIO‐12, Table S12).

The environmental niche reconstruction indicated that all tortoises and terrapins experienced periodic fluctuations in available area during the last 1 Mya and throughout the Holocene (Figure 1c, Figures S5–S22). The past mean area showed a wide variation among species, with a global average of 1.71 × 10^6^ km^2^. Aquatic and temperate climate species appeared to have a larger mean past suitable habitat extension than terrestrial and tropical climate species, respectively. The overall highest mean area availability was recorded during the last interglacial period with an average of 4.05 × 10^6^ km^2^ (Table S13).

### 3.4 Correlation Between ENM and PSMC

Overall, we observed no correlation between area availability and *N*_*e*_ fluctuations from the MIS19 interglacial to the last interglacial (*χ* ^2^ = 2.10, *p* = 0.15, phi = 0.46) and from LIG to LGM (*χ* ^2^ = 1.53 × 10^−31^, *p* = 1, phi = 0.18). No correlation was found when species were grouped either by habitat type or climatic zone (Table S14). A positive correlation was instead found between temperature fluctuation and demography changes through time from the MIS19 to the LIG (*χ* ^2^ = 4.55, *p* = 0.03) and from the LIG to the LGM (*χ* ^2^ = 18.18, *p* < 0.001). A significant correlation was also recorded for both tropical (*χ* ^2^ = 12.25, *p* < 0.001) and aquatic species (*χ* ^2^ = 14.22, *p* < 0.001) from the LIG to the LGM. No significant correlation was instead observed for either temperate or terrestrial species (Table S15).

### 3.5 Correlation Between Heterozygosity and Effective Population Size

**FIGURE 3:**
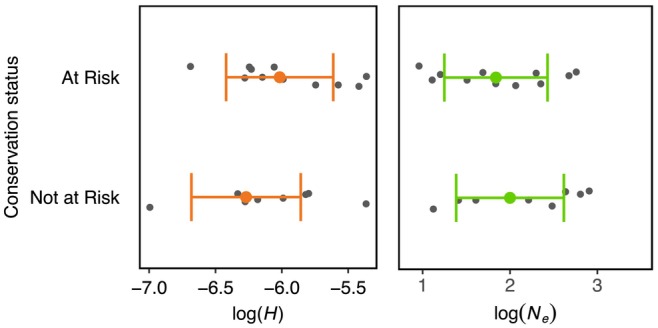
Heterozygosity (*H*) and mean effective population size (*N*_e_) estimated using a multiple regression model for ‘At risk’ (species listed as Vulnerable, Endangered and Critically Endangered according to IUCN criteria) and ‘Not at risk’ (species listed as Least Concern and Near Threatened). Orange and green dots and error bars represent model point estimates and 95% confidence intervals, respectively. Raw data points are shown in grey. The Tukey's post hoc honestly significant difference test found no significant difference among IUCN categories.

**FIGURE 4:**
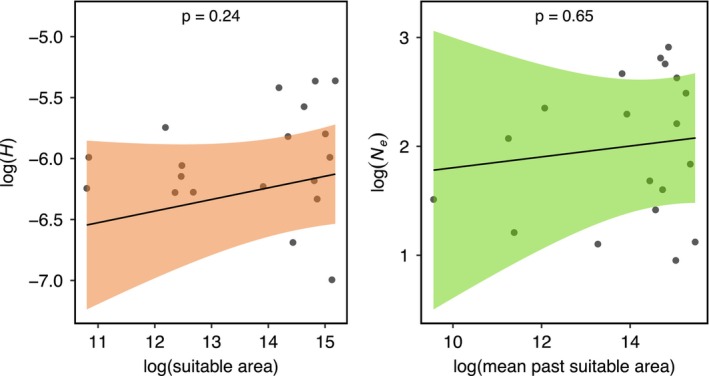
Relationships between current area availability and *H* and past mean area availability and mean *N*_*e*_ based on PGLS models. Lines and shaded areas represent model estimates and 95% confidence intervals, respectively. Raw data points are shown in grey.

The correlations between *H* and mean *N*_e_ (response variables), conservation status and suitable area availability were estimated using multiple regression models. We observed no significant correlation between conservation status and *H* or mean *N*_*e*_ (Figure 3, Figure S27). Similarly, we found no significant correlation between area availability and *H* or mean *N*_e_ (Figure 4). Significant correlation was instead found between current and past habitat availability and species conservation status (Figure S28).

## 4 Discussion

### 4.1 A Reference Genome for the European Pond Turtle

In this study, we produced a chromosome‐level reference genome for the European pond turtle *E. orbicularis* , the first to have a high‐quality genome characterised among all terrapins autochthonous to the European continent. The assembly, obtained using an integrated approach of long‐read HiFi sequencing, optical genome mapping and chromosome conformation capture, had a relatively low number of scaffolds and the highest N50, quality and completeness based on BUSCO scores of any of the currently published tortoise and terrapin genomes. Genome size was approximately 2.32 Gb, which falls within the range (1.87–2.57 Gb) recorded for the other 21 species of terrapins and tortoises considered in our study. We were able to physically map six reference scaffolds to karyotypic chromosomes using single‐chromosome sequencing techniques, a result rarely provided in assembled reference genomes (Lewin et al. 2019). This is of preliminary, significant importance since assemblies lacking a comprehensive physical assignment to chromosomes are usually ordered and named based on their size, and therefore lose information obtained by cytogenetic studies where pairing and ordering of chromosomes is generally based on the location of the centromere (Iannucci, Makunin, et al. 2021). Out of the 22 taxa we examined, only two species besides *E. orbicularis* ( *Chrysemys picta bellii* and *Trachemys scripta elegans* ) presented a physical mapping of part of their genome to the actual chromosomes. These, however, were produced using a bacterial artificial chromosomes approach, making the *E. orbicularis* genome the first to have chromosomes assigned using a ChromSeq technique.

Unlike flow sorting, micro‐dissection is a chromosome isolation approach that allows the isolation of only a few chromosome copies at a time. Sequencing micro‐dissected chromosomes usually results in very low coverage values, and data are not generally sufficient to proceed with a de novo assembly (Iannucci, Makunin, et al. 2021). Furthermore, the isolation approach using micro‐dissection is challenging, and chromosomes might break or get damaged during the process, making them difficult to sequence, which is why we managed to assign only part of the chromosomes in this study. The assignment of *E. orbicularis* genome to chromosomes will certainly benefit from the isolation of multiple copies of chromosomes via flow sorting in order to obtain a sufficient amount of sequencing data for de novo assembly of karyotypic chromosomes and subsequent comparison with scaffold‐based chromosomes.

### 4.2 Terrapin and Tortoise Demography and Ecology of the Past 10 million years

We performed a comparative analysis of tortoise and terrapin reference genomes to investigate patterns of demographic history in 22 species. Demographic trajectories estimated over the last 10 Mya showed recurrent cycles of population expansions and contractions as previously reported in Trionychidae and Geoemydidae (Liu et al. 2022; Ren et al. 2022). The earliest period from 10 Mya to the MIS19 (~787 kya) was characterised by initially stable effective population sizes which then began to decline concurrently with the Eocene–Oligocene transition and the formation of a permanent ice cap over the Antarctic region (Bartoli et al. 2005; Mudelsee and Raymo 2005). From the MIS19 to more recent times (~10 kya) we recorded a decline in *N*_e_, particularly as the temperature decreased at the end of the LIG (between 130 and 115 kya). Indeed, statistical testing showed that demographic fluctuations from the MIS19 to the LGM (~21 kya) were significantly correlated to an overall decrease in global surface temperature. Slightly different trends were recovered for species from different climatic zones. Temperate species showed an increase in *N*_e_ before the MIS19 while no deviation from the general pattern was observed in species from tropical zones. This difference could be attributed to adaptation to diverse climatic optima. Ectotherms from the tropics show a narrower thermal range and lower warming tolerance than species from temperate regions (Snyder and Weathers 1975; Clusella‐Trullas et al. 2011; Quintero and Wiens 2013; Bofill and Blom 2024). As a result, tropical species tend to have higher lethal minimum temperatures than temperate species (Moritz et al. 2012). Low temperatures probably led to a continued *N*_e_ decrease in tropical species and had a lower impact on temperate tortoises and terrapins, allowing for a larger fluctuation in *N*_e_ as a result of climatic changes.

Species from different habitats also differed in demographic trajectories. Terrestrial species had an overall lower *N*_e_ than aquatic species between the MIS19 and the LIG. Such differences could be explained by the differences observed between species adapted to different climatic conditions. Terrapins have a broader low thermal threshold than terrestrial species and can remain active at temperatures as low as 5°C (Brattstrom 1965). On the other hand, terrestrial species are more susceptible to harsher climates, having a mean thermal minimum of approximately 15°C, and fluctuations in temperature between interglacial and glacial periods may have had a stronger influence on their overall *N*_e_.

Among reptiles, a sharp decline in *N*_e_ between 100 and 10 kya was estimated in three families of crocodilians (Green et al. 2014), in varanids (Iannucci, Benazzo, et al. 2021), chameleons (Taft et al. 2023), beaded lizards (Dyson et al. 2022) and elapids (Ludington and Sanders 2021). A similar sharp decline was also recorded at the end of the last interglacial period for teleost species (Li et al. 2021). An overall decrease in environmental temperatures (van de Wal et al. 2011) can be particularly challenging for ectothermic species and may have contributed to the observed demographic reductions at the end of the last interglacial period. While similar patterns of demographic decline were described in vertebrates, these trends cannot be attributed solely to temperature changes. In birds, for instance, *N*_e_ fluctuations were not confined to the end of the last glacial period, and some species even experienced a population increase between 100 and 10 kya (Kozma et al. 2016; Germain et al. 2023). Moreover, both birds and mammals often had *N*_e_ linked to factors such as habitat availability and life history traits, including body size and diet (Lorenzen et al. 2011; Chattopadhyay et al. 2019; Brüniche‐Olsen et al. 2021; Germain et al. 2023). Unlike turtles, birds and mammals, being endotherms and more vagile, could probably better adapt to temperature fluctuations and mitigate the effects of environmental changes on population survival.

PSMC provides a robust approach for estimating *N*_e_ over distant time periods (Li and Durbin 2011), however, it relies on assumptions that may bias estimates of actual population size for very recent demographic trends (Leroy et al. 2021; Nadachowska‐Brzyska et al. 2022). Moreover, under‐ or overestimated values of mutation rate and generation time can skew the graph, moving the curve along the axis but keeping the principal shape of the *N*_e_ fluctuation. PSMC also assumes panmixia and absence of population structure (Mazet et al. 2016; Nadachowska‐Brzyska et al. 2022). Although coalescent methods may not provide accurate estimates of *N*_e_ fluctuation over time, the PSMC used in our study nevertheless allowed construction of a comparative framework outlining an overall pattern of past demographic trends (Li et al. 2025).

### 4.3 Habitat Fluctuations During the Pleistocene

Over the past 1 Mya, the extent of available habitat was subject to alternate periods of contraction and expansion which differed greatly among species, latitude and habitat type (Hofreiter and Stewart 2009). Species living at higher latitudes (temperate species) experienced a significant reduction in available area after the end of the last interglacial period and an increase in suitable habitat after the end of the last glacial maximum (Table S13, Figures S11–S26). On the other hand, lower latitude species (tropical species) experienced a lower degree of change or even an increase in available suitable area after the last interglacial period (Table S13, Figures S11–S26). The niche models including all 19 bioclimatic variables indicated the minimum temperature of the coldest month as the primary determinant of suitable habitat for the majority of tortoises and terrapins except for riverine species, for which precipitation‐related variables were the most important. According to the second set of models including 14 variables, precipitation‐related variables were particularly important for all species of terrapins, while temperature‐related variables remained predominant for all species of tortoises. Temperature and precipitation appeared to affect the distribution of suitable habitat in tortoises and terrapins, respectively, not only during the Pleistocene but also in the Cretaceous and Eocene (Waterson et al. 2016; Chiarenza et al. 2023). During the mid‐Cretaceous, freshwater species had a wide distribution ranging from low polar latitudes to the equator. A shift towards mid to low latitudes occurred during the late Cretaceous, followed by a further expansion of suitable habitats to higher latitudes in the late Eocene. Tortoises appeared to have maintained a more localised and reduced distribution of suitable habitats in the tropical zone and at mid‐low latitudes of the southern hemisphere (Chiarenza et al. 2023). Starting from the late Eocene, a drier and cooler climate (Francis et al. 2008) led to a latitudinal shift towards the equator of the distribution of suitable areas of both freshwater and terrestrial species. By the end of the Eocene, terrapins and tortoises were distributed in a variety of biomes thanks to favourable climatic conditions and adaptation to new available habitats (Chiarenza et al. 2023).

The reconstruction of the amount of suitable area across time described in our study falls within the niche limits presented by Chiarenza et al. (2023) and follows a similar pattern of latitudinal increase and reduction of suitable habitat during interglacial and glacial periods, respectively. Wet and warm environmental conditions during the MIS19, last interglacial and early Holocene led to an increase in suitable area availability for freshwater species at higher latitudes. On the other hand, the decrease in temperature after the last interglacial resulted in a reduction of suitable habitat and a range shift towards the equator. Terrapins and tortoises from tropical ecosystems were differentially affected by a relatively colder environment, for some species were subject to an increase of suitable area due to a reduction in eustatic sea level and emergence of new landmasses, while others experienced a reduction of suitable area due to low rainfall recorded at low latitudes during the last glacial period (Gasse 2000). Indeed, as described for the period spanning from the Cretaceous to the late Eocene, warm and dry conditions can significantly affect the distribution of those freshwater species which are capable of withstanding warmer climates than the present‐day average, with precipitations being an important limiting factor (Chiarenza et al. 2023). Even though no correlation was found between habitat availability and *N*_*e*_ fluctuations as in other groups of vertebrates (Chattopadhyay et al. 2019; Brüniche‐Olsen et al. 2021), the common trend of demographic decrease recorded for tortoises and terrapins after the transition to colder climates advocates the significant influence environmental variables had on reptiles during the latest geologic epochs.

Past range distribution and ENM are not devoid of technical challenges (Warren 2012). Geographical sample bias due to site accessibility (sites closer to cities and roads often show a relatively higher sampling density), land use and urbanisation is one of the most common issues in niche modelling. For instance, the absence of species occurrences in areas that are climatically favourable but occupied by human activities can cause misestimations of the actual environmental niche distribution (Ay et al. 2017; De Souza and Vasconcelos 2018). That is not easy to overcome, however, all species considered in our study appear to occur also in areas which are usually difficult to access (e.g., Amazonian forest and South Asian rainforests). This is true particularly for freshwater terrapins, which are generally easy to spot in rivers and river banks.

### 4.4 Genomic Diversity, Conservation Status and Suitable Area

Heterozygosity and effective population size are indicators of current and past genomic diversity and important parameters to reconstruct demographic histories (Kanaka et al. 2023). The use of genetic diversity information in IUCN Red List status assessments has been advocated to better inform the conservation status of threatened and understudied species and establish a link between genomic diversity indicators and IUCN categories for inference of the conservation status for data deficient or yet to be assessed taxa (Vitorino et al. 2019; Garner et al. 2020; Schmidt et al. 2023). Studies on birds and mammals showed that genomic diversity indicators such as heterozygosity, runs of homozygosity (ROH) and *N*_e_ were correlated to IUCN Red List status and could therefore be used to assess the conservation status of data deficient and not yet assessed species (Brüniche‐Olsen et al. 2019, 2021; Vitorino et al. 2019; Wilder et al. 2023). We assumed that tortoises and terrapins classified as Vulnerable, Endangered and Critically Endangered in the IUCN Red List would show lower genomic diversity and effective population size compared to Least Concern or Near Threatened species. Similarly, genomic diversity and *N*_e_ would be positively correlated with the extent of suitable area. Nevertheless, the PGLS analysis found no significant relationships between heterozygosity, average *N*_e_, mean available suitable area and threat status. The significant relationship found between habitat availability and conservation status was expected since the IUCN conservation assessments are mainly based on available habitat and habitat encroachment (IUCN 2012). Terrapins and tortoises are particularly long‐lived species and have longer generation times, so that the genomic signature of population decline due to recent habitat loss and fragmentation may not yet be distinguishable (Clark et al. 2024). Several generations may, in fact, be necessary for a decrease in areas of suitable habitats to affect genomic diversity, so that the level of threats based on ecological and biogeographical data may not mirror current genomic profiles (Amos and Balmford 2001; McCoy et al. 2010; Pinto et al. 2024). Additionally, the lack of correlation between effective population size, threat status and suitable area availability could be explained by demographic history if recent population decline came after a reduction in *N*_e_ during the Pleistocene (Nadachowska‐Brzyska et al. 2015; Brüniche‐Olsen et al. 2019). No correlation between threat status and genetic diversity was found for several other species of vertebrates including fish, birds and mammals (Brüniche‐Olsen et al. 2018; Martinez et al. 2018; Schmidt et al. 2023). Different results are therefore being produced on the extent to which genetic parameters can aid the assessment of conservation status and indication of threatened categories defined by IUCN Red List criteria, particularly for data deficient taxa. Although genomic investigations may not always be consistent indicators of threat status, such is the case for terrapins and tortoises, assessments of which genomic parameter could best serve this purpose should be supported. Eventually, genomic metrics will need to be considered important measures of key components of biodiversity change and proposed as essential biodiversity variables (Jetz et al. 2019) to support species vulnerability and resilience assessment and inform conservation policy decisions.

## Author Contributions

M.S., A.I. and C.C. conceived the project. M.B. and C.N. provided and prepared samples for genomic analysis of *Emys orbicularis* , J.B., N.J. and J.M. carried out genome sequencing of *E. orbicularis* , B.K. and J.M. were involved in data wrangling, A.I. performed chromosome micro‐dissection and sequencing with assistance from C.N. and V.T., M.S. assembled the genome of *E. orbicularis* and performed data analysis. M.P.F. curated the genome assembly. M.S., A.I. and C.C. wrote the manuscript. A.B.‐O., G.F., G.C., S.F., E.D.J. and D.N. provided feedback and input on manuscript content and analyses. All authors approved the final manuscript.

## Disclosure

Benefit‐sharing statement: Italy has yet to ratify the Nagoya protocol. However, access to genetic resources and the fair and equitable sharing of benefits arising from their utilisation have been duly considered as far as the generation of genomic data for *Emys orbicularis* concerns. Co‐authors of this manuscript were directly involved in sample collection and are part of the local community where sampling occurred. Collection permits were provided by the Italian Ministero dell'Ambiente e della Tutela del Territorio e del Mare (Protocol PNM‐2011‐0020019‐28/09/2011). Genomic resources generated by this study are accessible on public databases and can be used by the relevant parties for research, development and conservation of the European pond turtle in central Italy.

## Conflicts of Interest

The authors declare no conflicts of interest.

## Supporting information


**Figures S1–S28:** men70040‐sup‐0001‐Figures.pdf.

**Tables S1–S15:** men70040‐sup‐0002‐Tables.xlsx.

## Data Availability

The genome assembly and the HiFi and Hi‐C raw read data were deposited in the National Center for Biotechnology Information (NCBI) with BioProject number PRJNA919152. Optical maps are available in GenomeArk at the URL: https://www.genomeark.org/vgp‐all/Emys_orbicularis.html. The Illumina read sequences of the micro‐dissected chromosomes are deposited in NCBI with BioProject number PRJNA919152. The alternate haplotype is deposited in the National Center for Biotechnology Information (NCBI) with BioProject number PRJNA919101. Dataset Keys of the occurrence records used in the models can be found at https://doi.org/10.15468/dd.rbr6ff. All scripts and a complete occurrence dataset are available in GitHub at: https://github.com/msozzoni/DemographicDynamicsInTortoisesAndFreswhaterTurtles.
