# Supplementary material for: Quaternary Habitat Fluctuations and Demographic Dynamics in Turtles Inferred From Environmental Niche Modelling and Whole Genome Data
Source: Mol Ecol Resour. 2025 Sep 4;25(8):e70040. doi: 10.1111/1755-0998.70040 (PMC12550490; doi:10.1111/1755-0998.70040)
Supplement: Supplementary file 1 — Figures S1–S28: men70040‐sup‐0001‐Figures.pdf. [file MEN-25-e70040-s002.pdf]

MOLECULAR ECOLOGY RESOURCES

Supplemental Information for:

Quaternary habitat fluctuations and demographic dynamics in tortoises and freshwater turtles inferred from environmental niche modelling and whole genome data

Marcella Sozzoni[1] | Jennifer Balacco[2] | Massimo Bellavita[3]* | Anna Brüniche-Olsen[4] | Giulio Formenti[2,5,6] | Nivesh Jain[2] | Bonhwang Koo[2] | Jacquelyn Mountcastle[2] | Marc Palmada-Flores[7] | Vladimir Trifonov[8] | Guido Chelazzi[1] | Sara Fratini[1] | Erich D. Jarvis[2,5,6] | Chiara Natali[1] | Davide Nespoli[1] | Claudio Ciofi[1] | Alessio Iannucci[1]

1 Department of Biology, University of Florence, Sesto Fiorentino, Italy
2 Vertebrate Genome Laboratory, The Rockefeller University, New York, NY, USA
3 Riserva Naturale Regionale Monte Rufeno, Acquapendente (VT), Italy
4 Center for Macroecology, Evolution and Climate, University of Copenhagen, Copenhagen, Denmark
5 Laboratory of Neurogenetics of Language, The Rockefeller University, New York, NY, USA
6 Howard Hughes Medical Institute, Chevy Chase, MD, USA
7 Tree of Life, Wellcome Sanger Institute, Cambridge, United Kingdom
8 Institute of Molecular and Cellular Biology SB RAS, Novosibirsk, Russia

* Current address: Riserva Naturale Regionale Selva del Lamone, Farnese (VT), Italy

Table of contents

European pond turtle genome statistics .... page 2
Demographic and Area availability reconstruction .... page 6
Conservation status correlations .... page 28

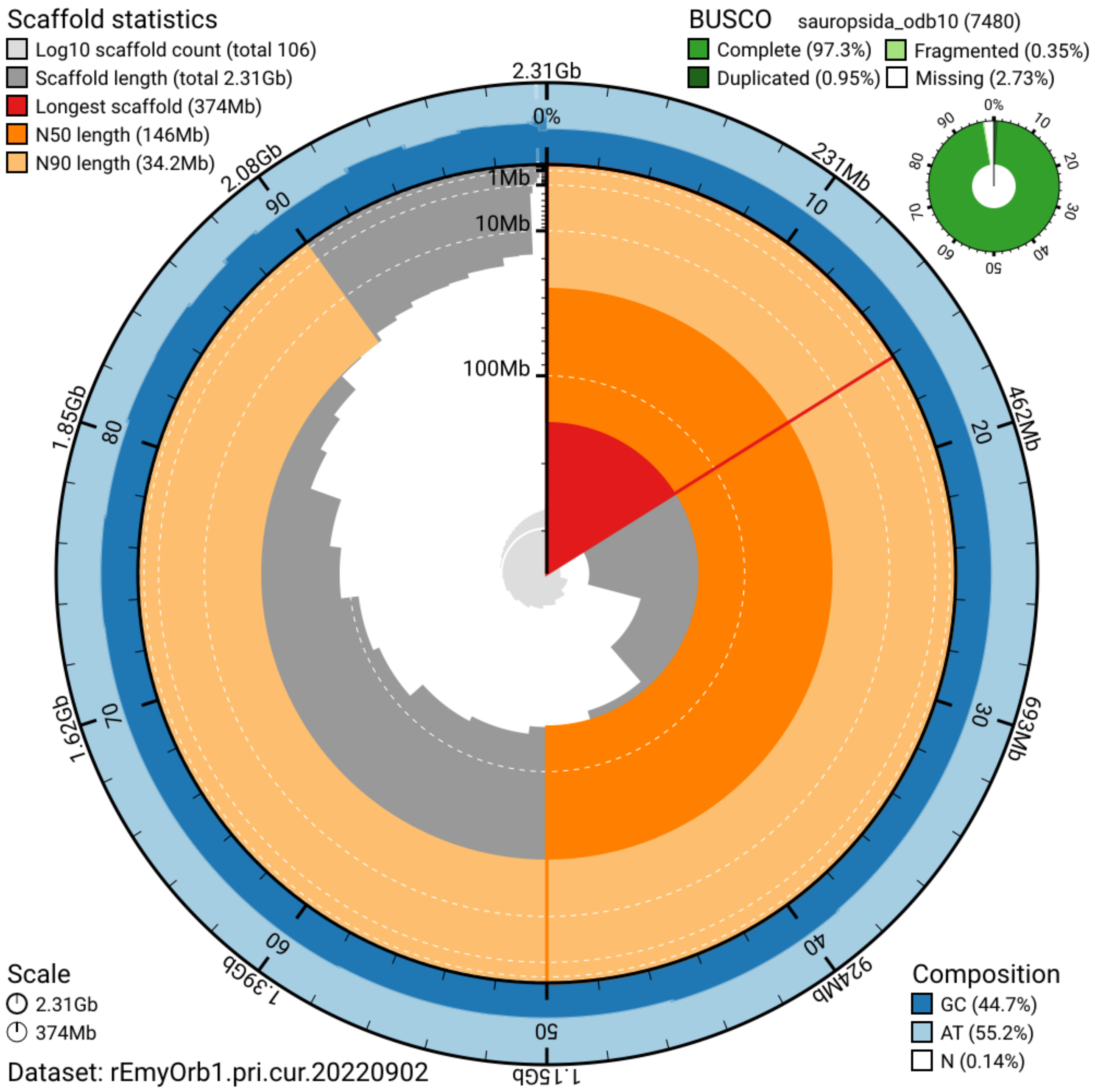

Figure S1. Genome assembly metrics for *Emys orbicularis,* rEmyOrb1.hap1. The central BlobToolKit Snailplot shows a summary of assembly quality metrics and BUSCO gene completeness statistics. The distribution of scaffold lengths is reported in grey with the plot radius scaled to the longest scaffold (373,000,000 bp, shown in red). Orange and pale-orange arcs show the N50 and N90 scaffold lengths (150,000,000 and 34,000,000 bp, respectively). The pale grey spiral in the middle shows the cumulative scaffold count on a log scale with a white scale line indicating successive orders of magnitude. The dark and light blue areas around the perimeter of the plot show the distribution of GC and AT content at intervals each representing 0.1% of the 2,324,111,602 bp assembly. The white regions represent the proportion of Ns in the assembly. A summary of BUSCO genes in the sauropsida_odb10 dataset is shown at the top right.

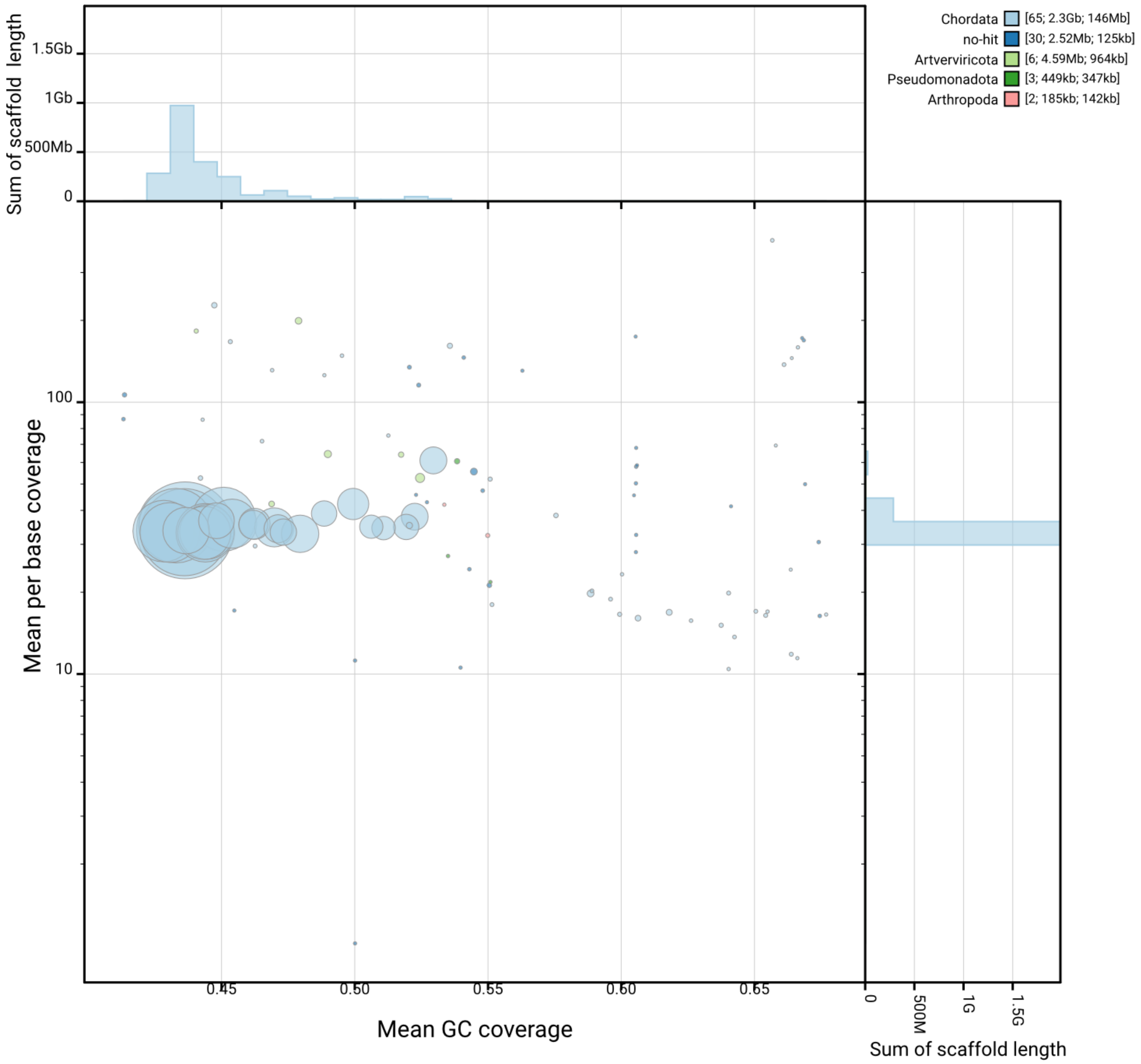

Figure S2. Genome assembly metrics for *Emys orbicularis* shown by the BlobToolKit GC-Coverage plot. The sum of scaffold length reported at the top of the graph relates to the mean GC content, while the sum of scaffold length to the right of the main graph refers to the mean per-base coverage. Scaffolds are coloured by phylum and circles are sized in proportion to scaffold length.

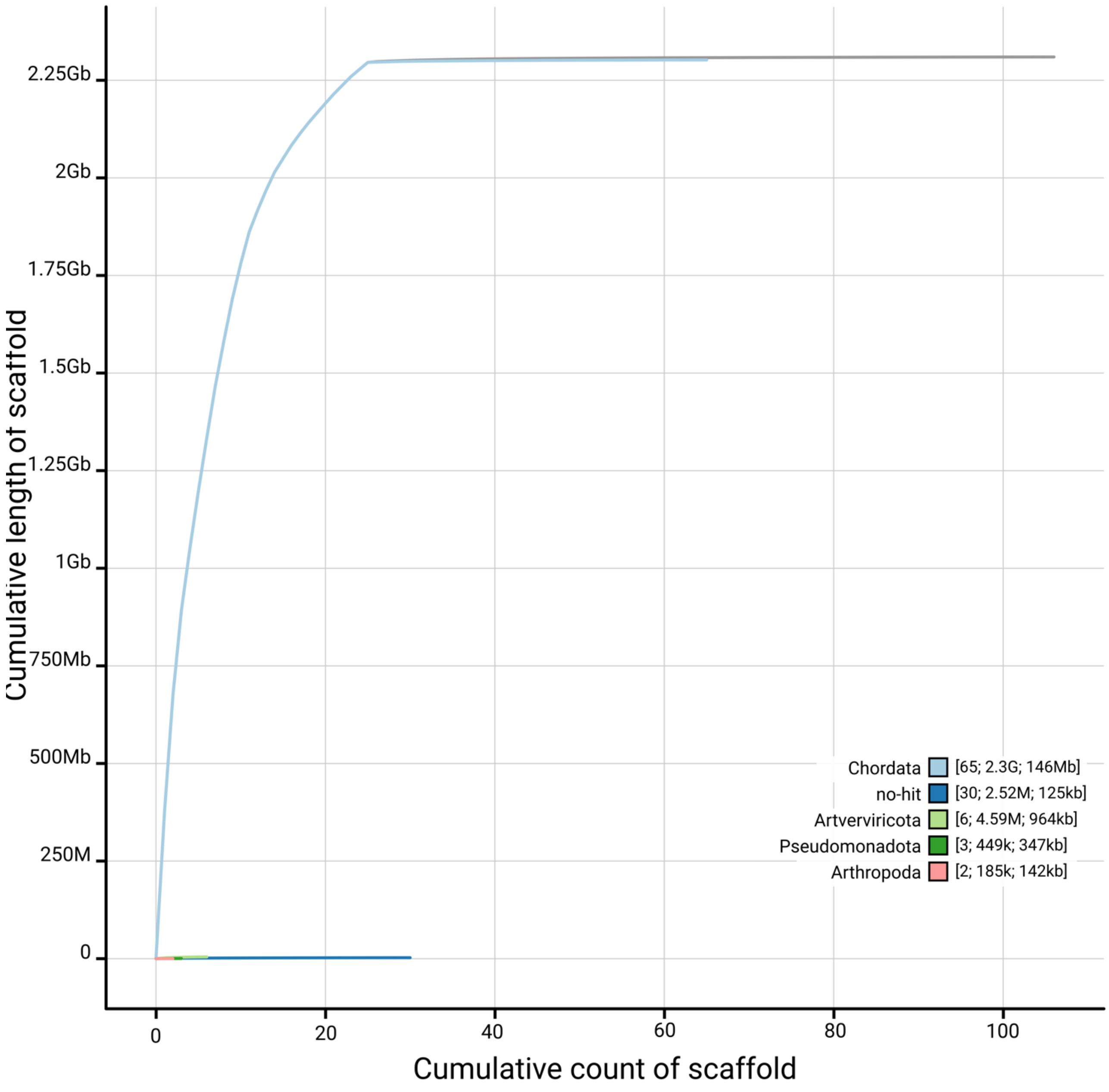

Figure S3. Genome assembly metrics for *Emys orbicularis* shown by the BlobToolKit cumulative sequence plot. Coloured lines show cumulative lengths of scaffolds assigned to each phylum using the BUSCO genes taxrule. The grey line shows cumulative length for all scaffolds.

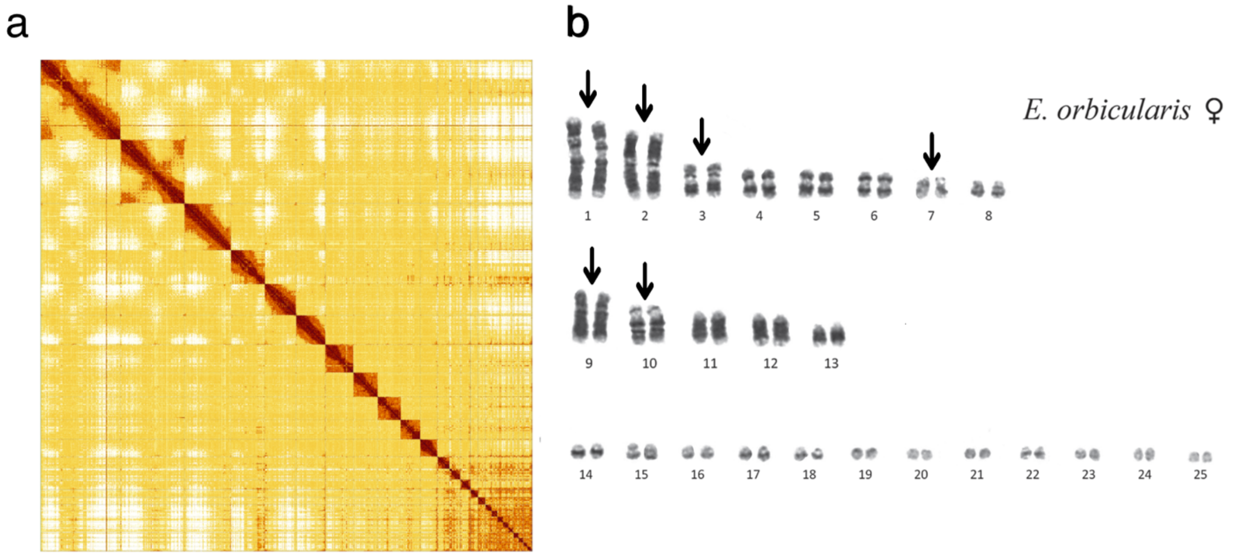

Figure S4. Hi-C contact map and chromosomes assignment in *Emys orbicularis*. (a) Hi-C contact map of the rEmyOrb1.hap1 assembly shown in PretextView. Chromosomes are arranged by size from left to right and top to bottom. (b) Karyotype of *E. orbicularis* (Iannucci et al., 2019). Arrows indicate the chromosomes that were assigned to six scaffolds of the reference genome using a ChromSeq approach.

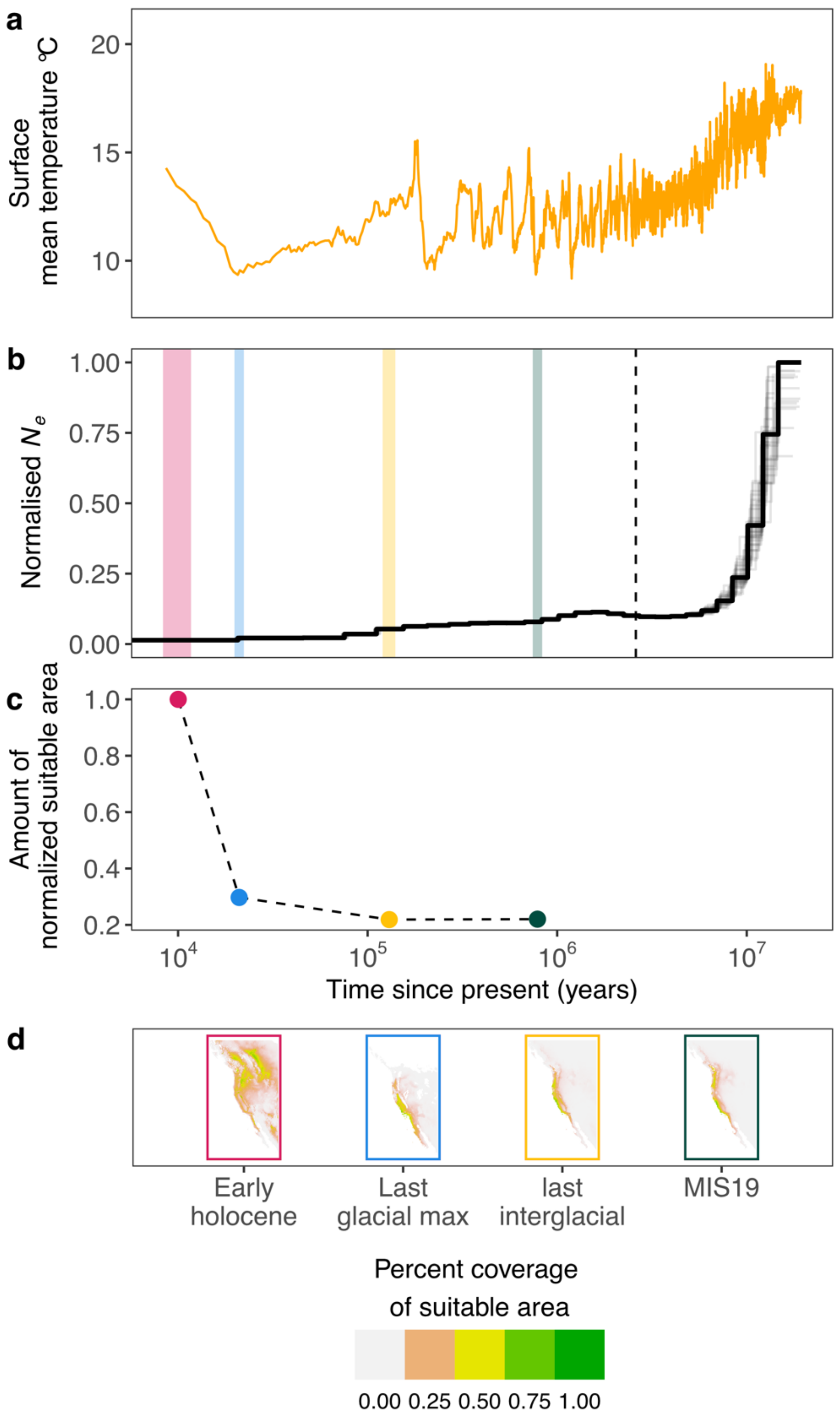

Figure S5. Effective population size trajectories over time and Environmental Niche Modelling (ENM) for *Actinemys marmorata.* The orange line in (a) represents the mean surface temperature estimated for the last 10 Mya. A pairwise sequentially Markovian coalescent (PSMC) was used in (b) to estimate N_e variation over the past 10 Mya. The black line shows N_e values over time and light grey lines represent PSMC bootstrap replicates. Coloured vertical bars correspond, from left to right, to the Early Holocene, Last Glacial Maximum, Last Interglacial, and Marine Isotope Stage 19 Interglaciation (MIS19). The dashed line represents the first appearance of a permanent Antarctic ice cap. Extent (c) and percent coverage (d) of suitable area estimated using ENM are reported from 1 Mya to 10 Kya. Values of N_e and suitable area were normalised by dividing all values by the maximum value estimated over the four time periods.

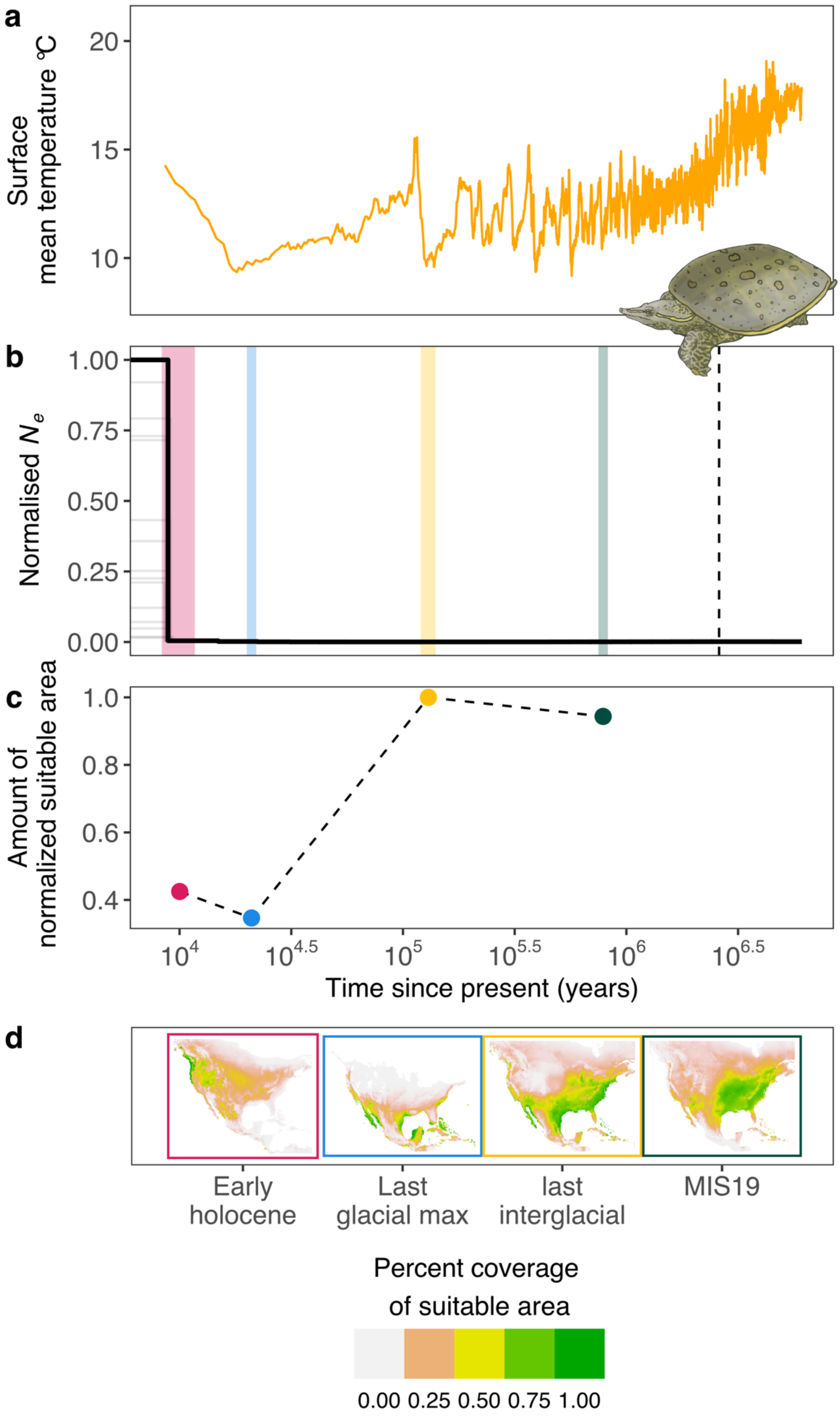

Figure S6. Effective population size trajectories over time and Environmental Niche Modelling for *Apalone spinifera*. Details as in Figure S5.

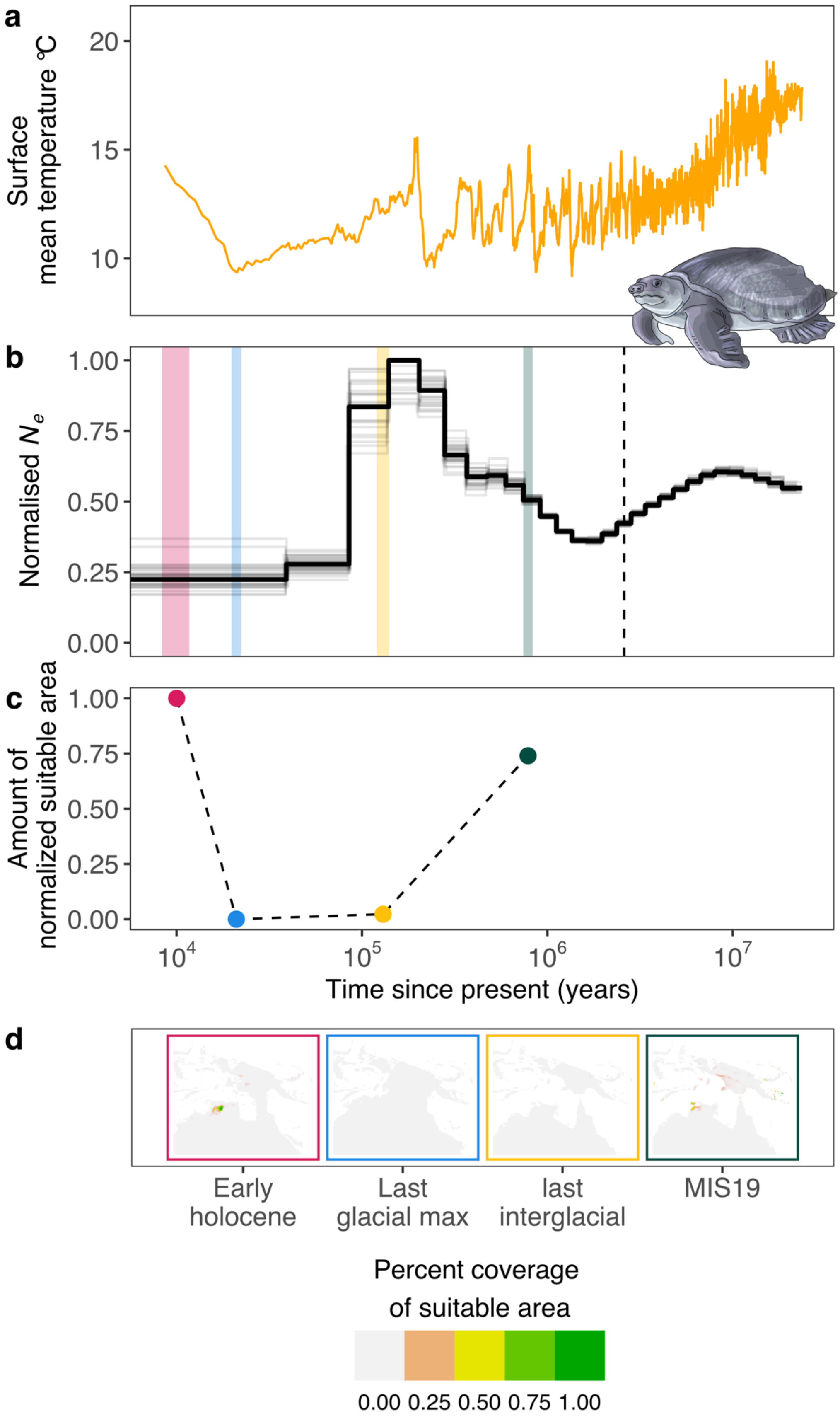

Figure S7. Effective population size trajectories over time and Environmental Niche Modelling for *Carettochelys insculpta.* Details as in Figure S5.

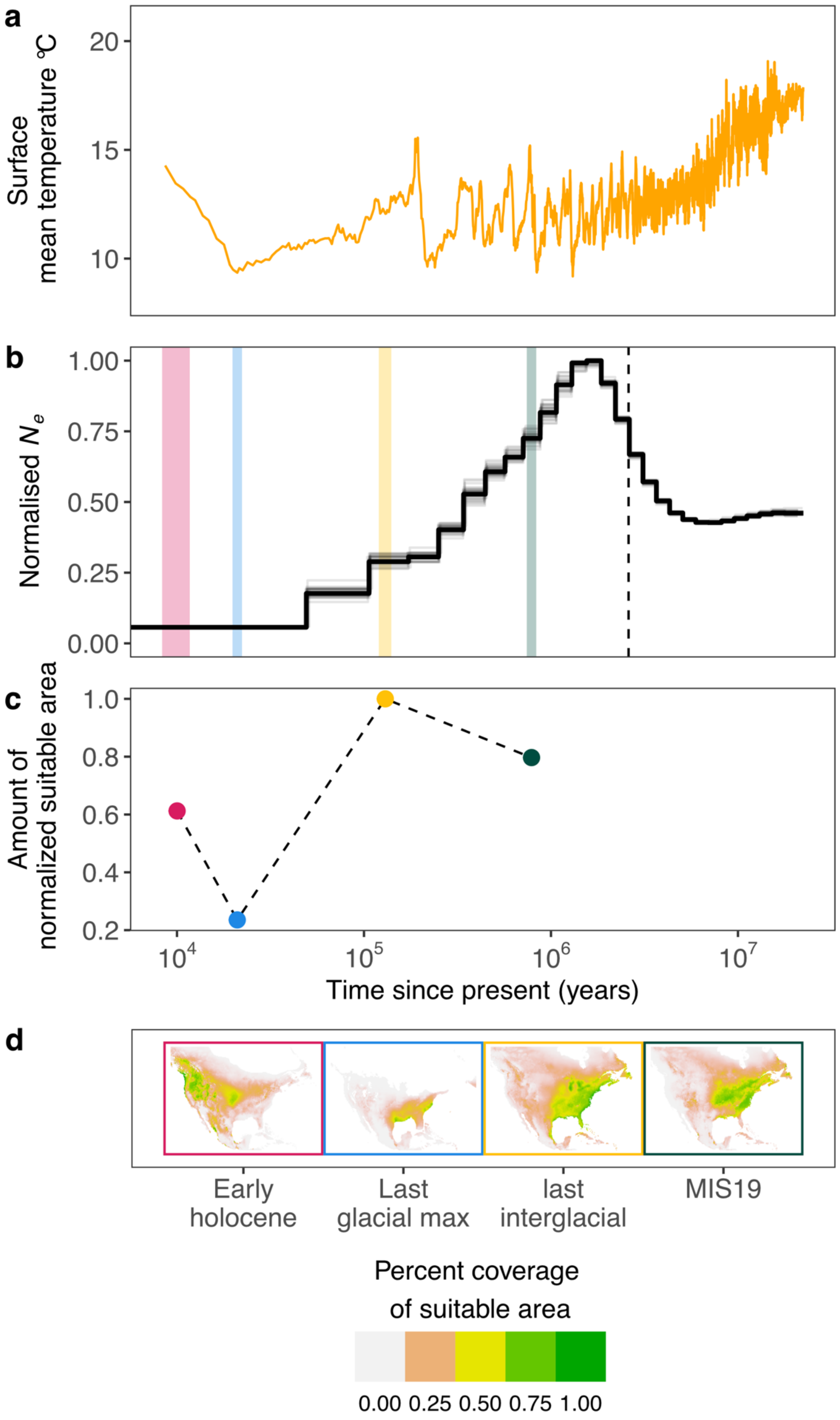

Figure S8. Effective population size trajectories over time and Environmental Niche Modelling for *Chelydra serpentina.* Details as in Figure S5.

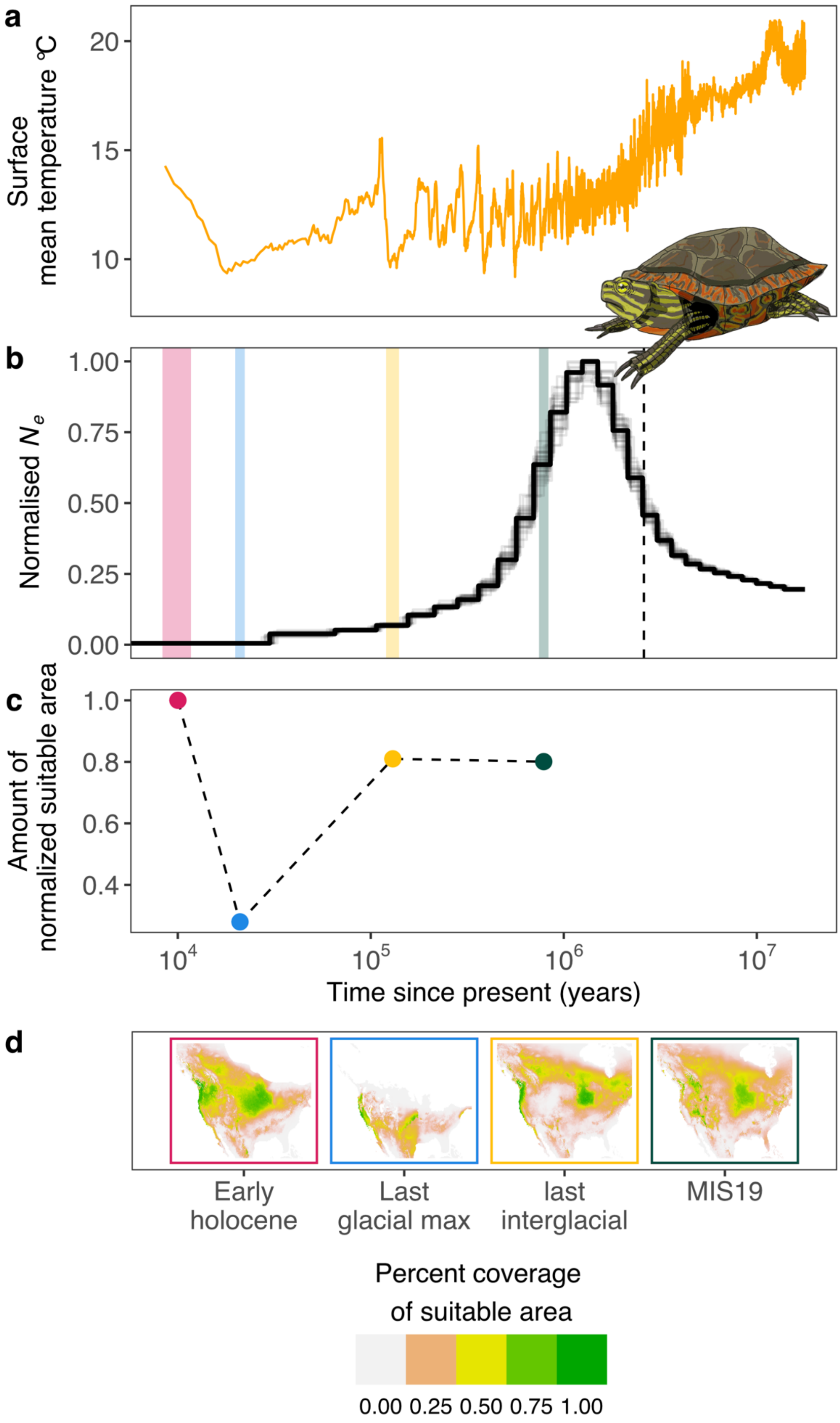

Figure S9. Effective population size trajectories over time and Environmental Niche Modelling for *Chrysemys picta bellii.* Details as in Figure S5.

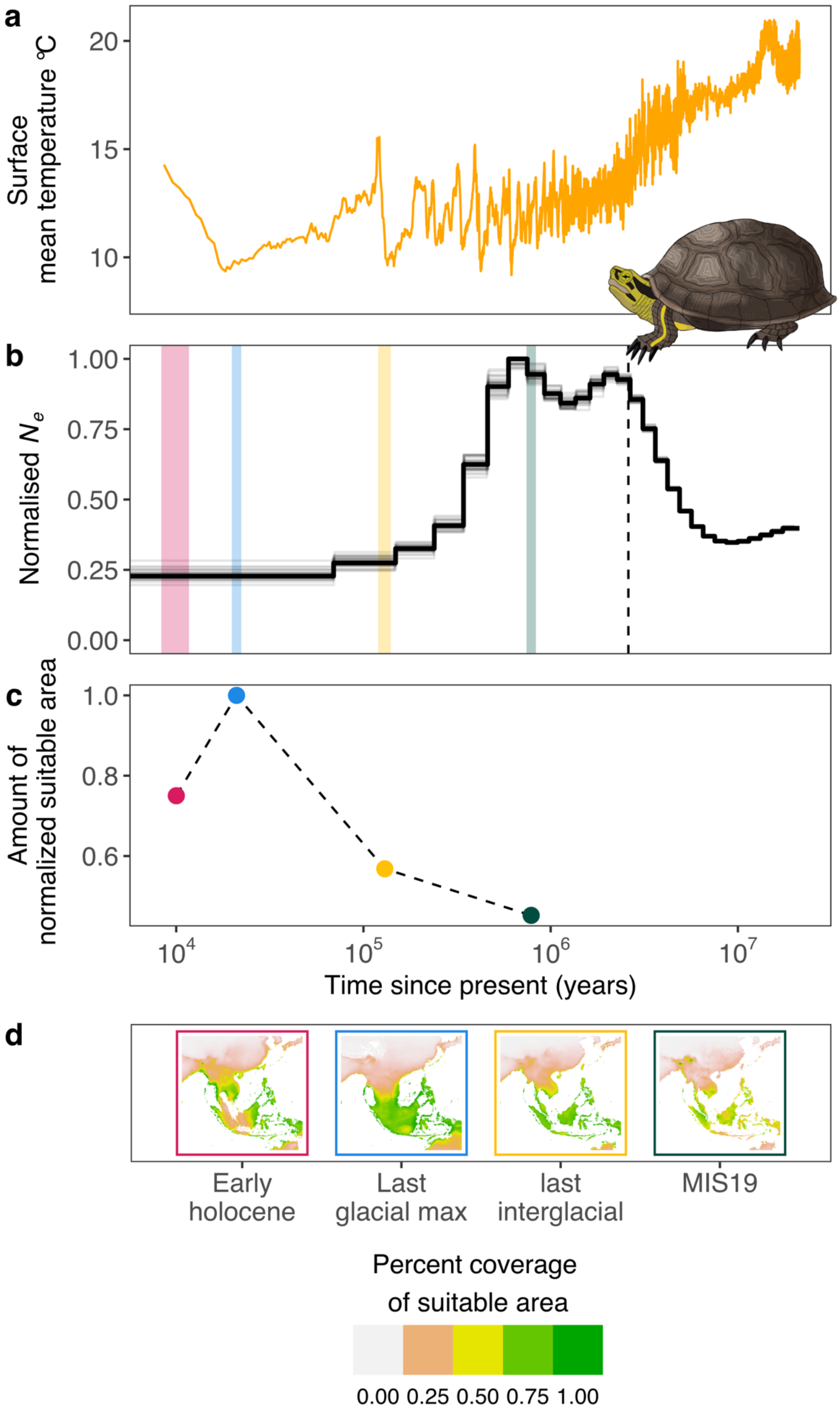

Figure S10. Effective population size trajectories over time and Environmental Niche Modelling for *Cuora amboinensis.* Details as in Figure S3.

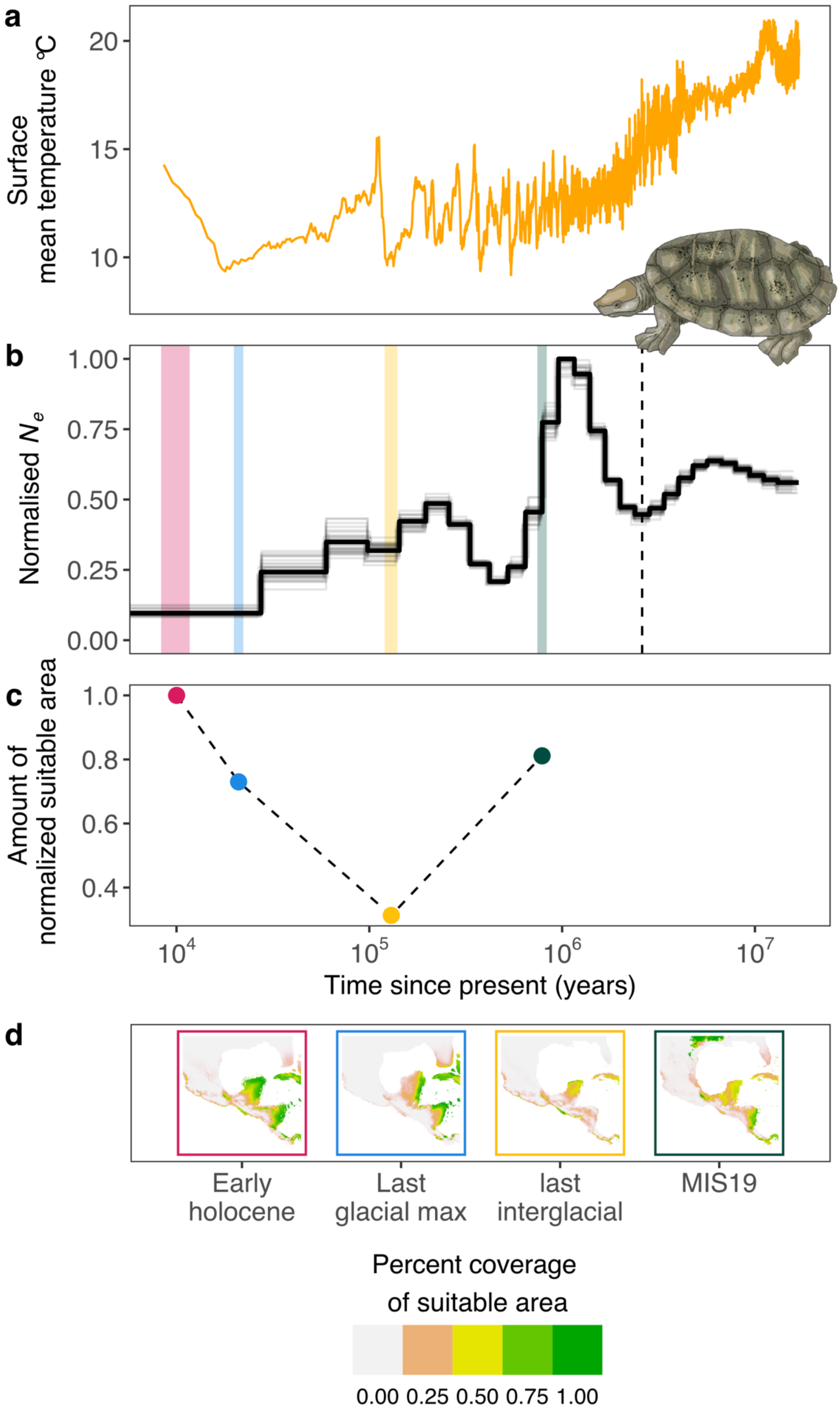

Figure S11. Effective population size trajectories over time and Environmental Niche Modelling for *Dermatemys mawii.* Details as in Figure S3.

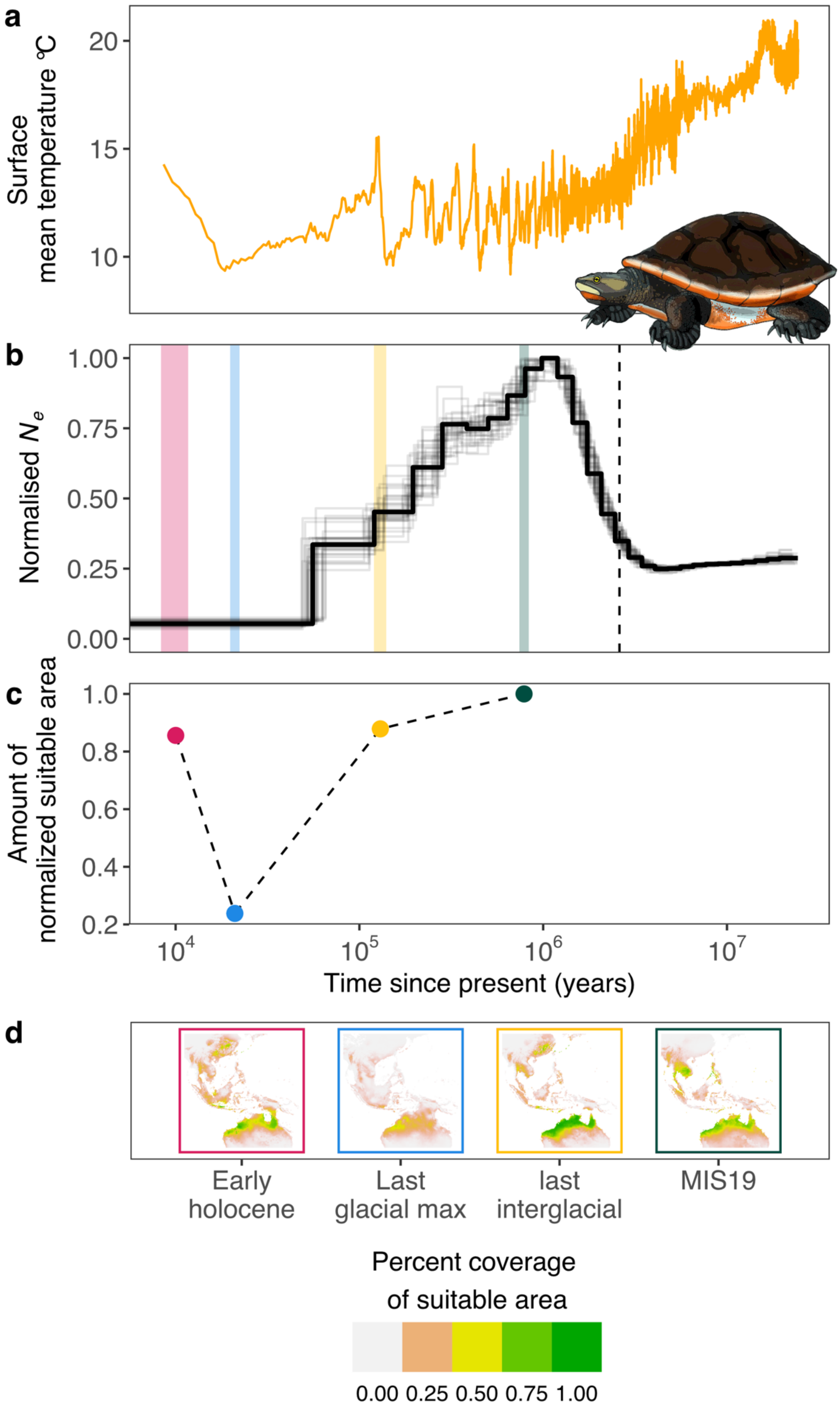

Figure S12. Effective population size trajectories over time and Environmental Niche Modelling for *Emydura subglobosa.* Details as in Figure S5.

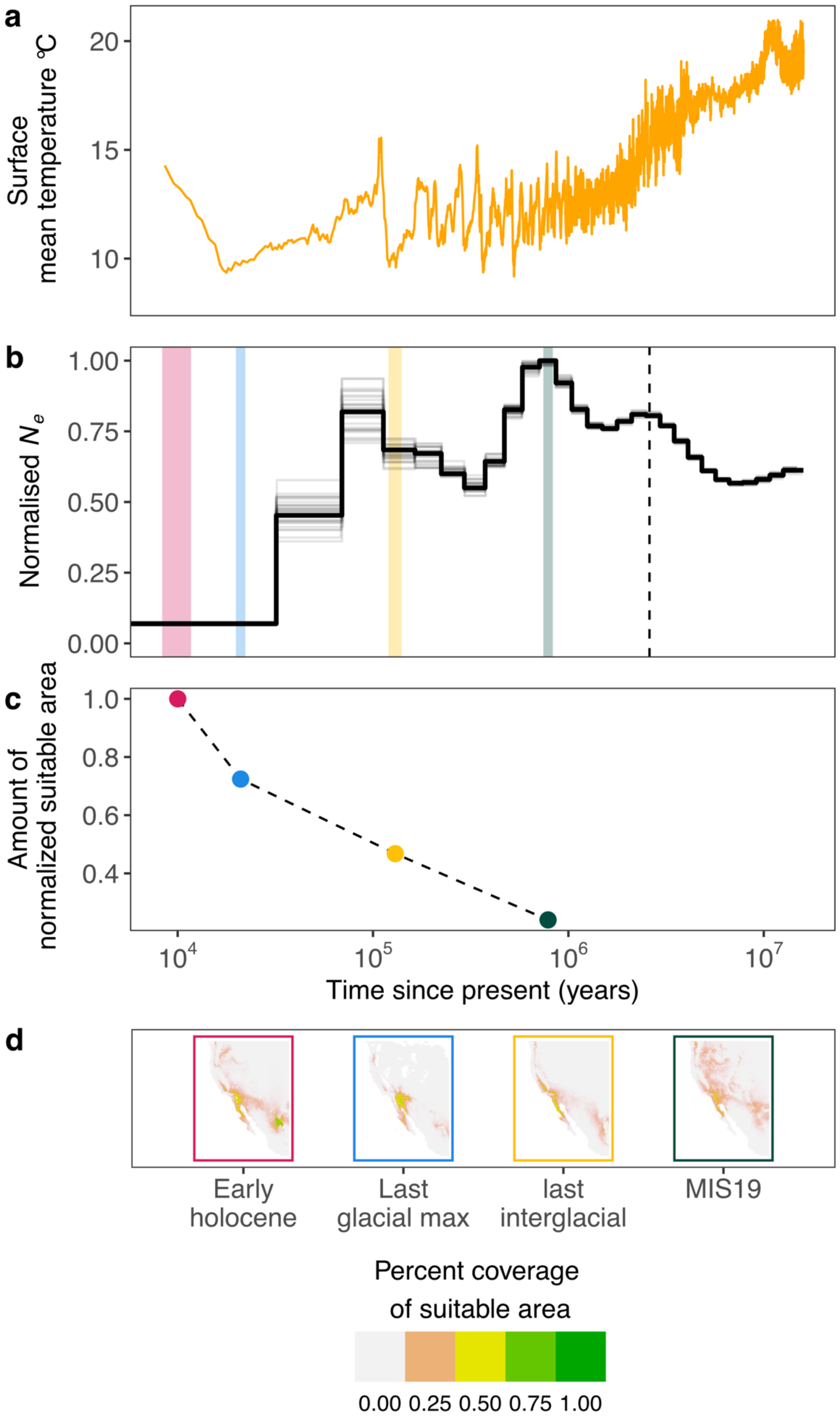

Figure S13. Effective population size trajectories over time and Environmental Niche Modelling for *Gopherus agassizii.* Details as in Figure S5.

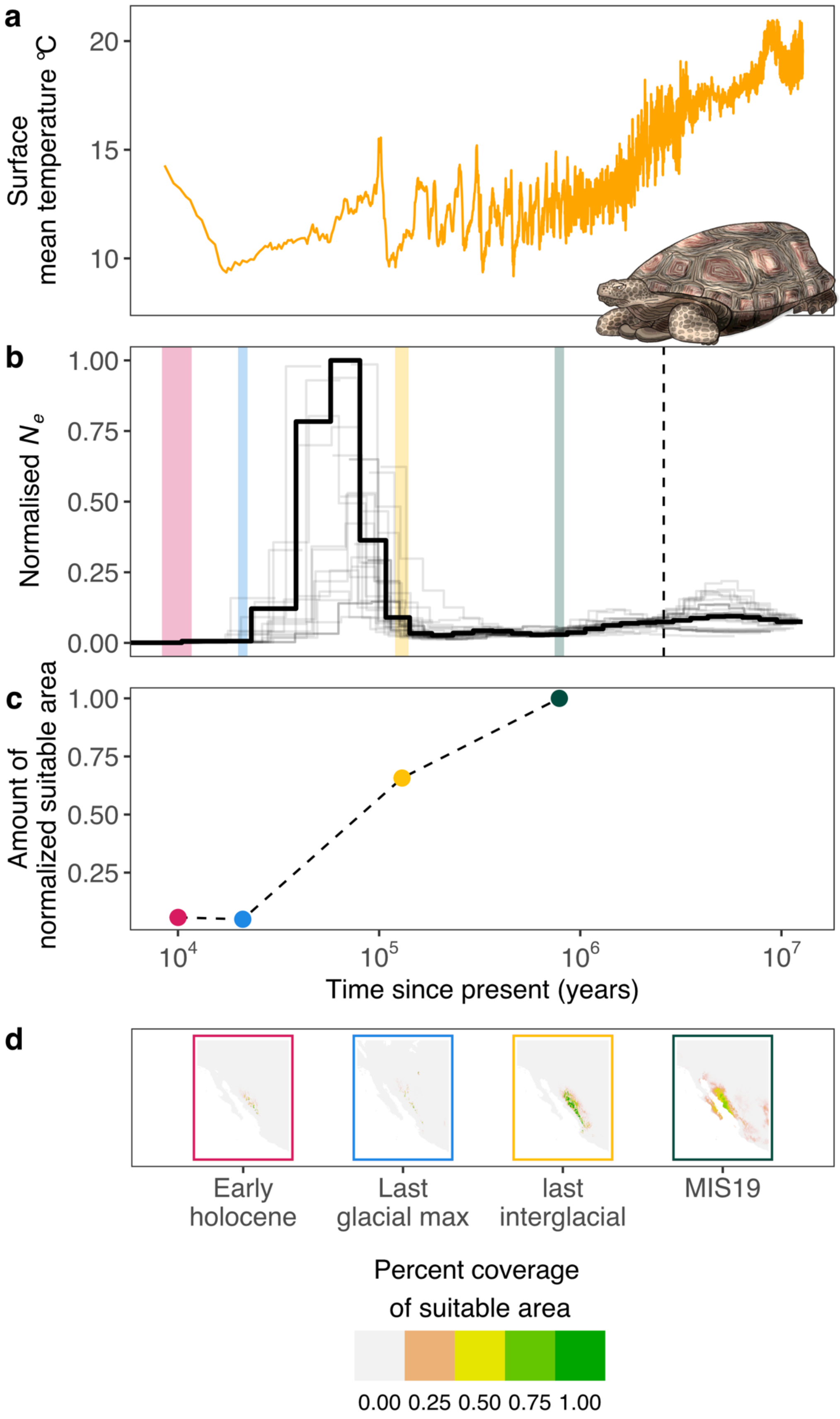

Figure S14. Effective population size trajectories over time and Environmental Niche Modelling for *Gopherus evgoodei.* Details as in Figure S5.

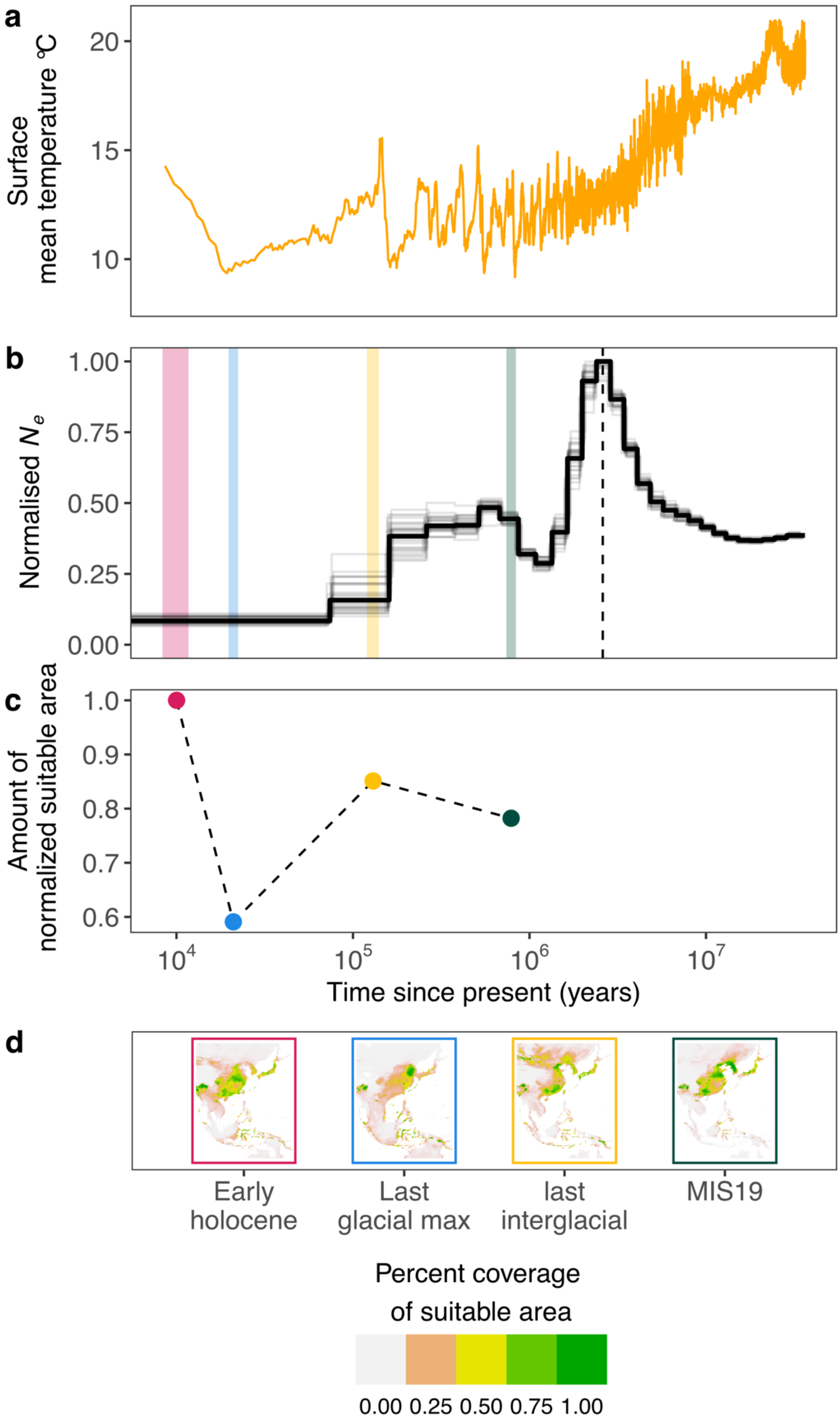

Figure S15. Effective population size trajectories over time and Environmental Niche Modelling for *Mauremys reevesii.* Details as in Figure S5.

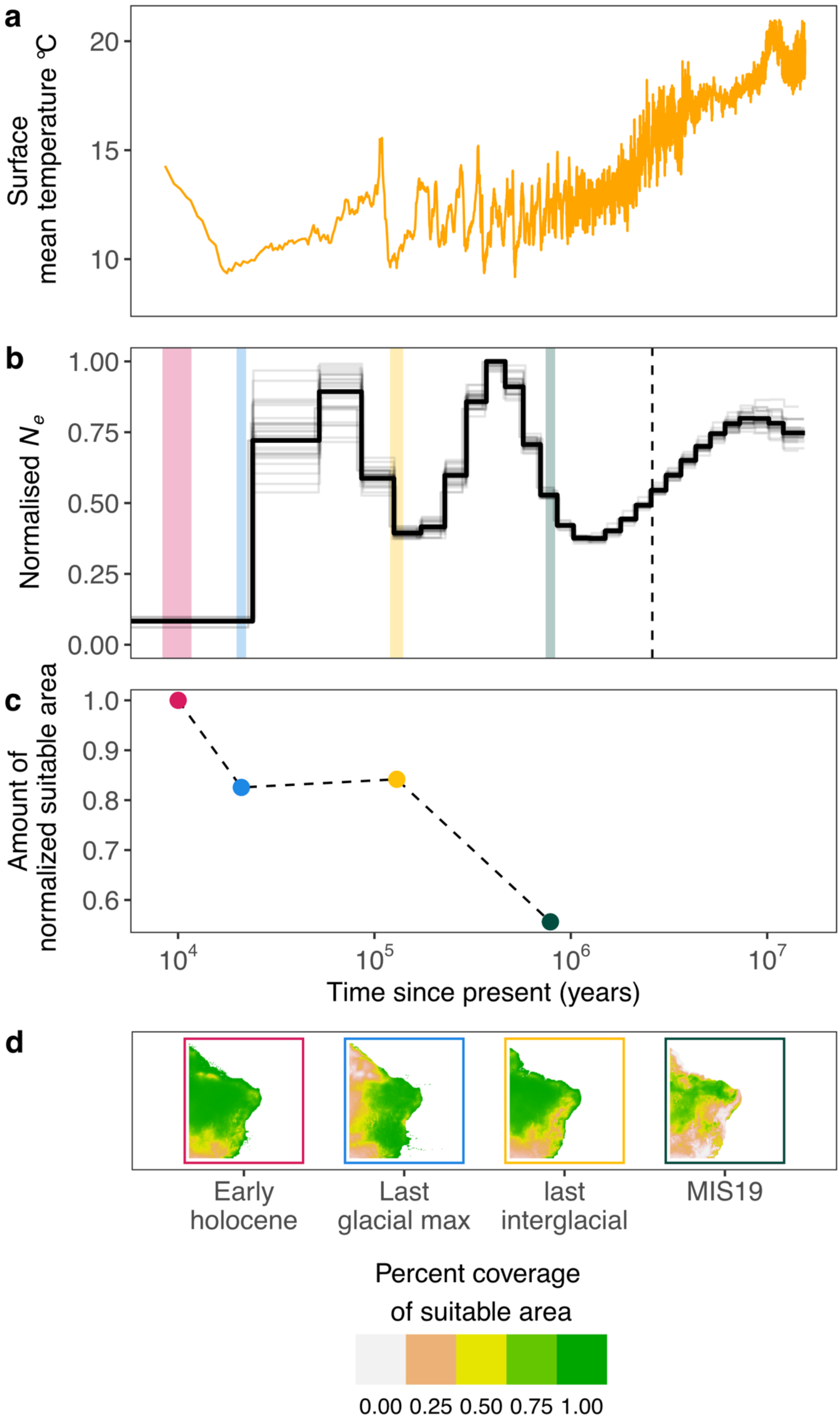

Figure S16. Effective population size trajectories over time and Environmental Niche Modelling for *Mesoclemmys tuberculate.* Details as in Figure S5.

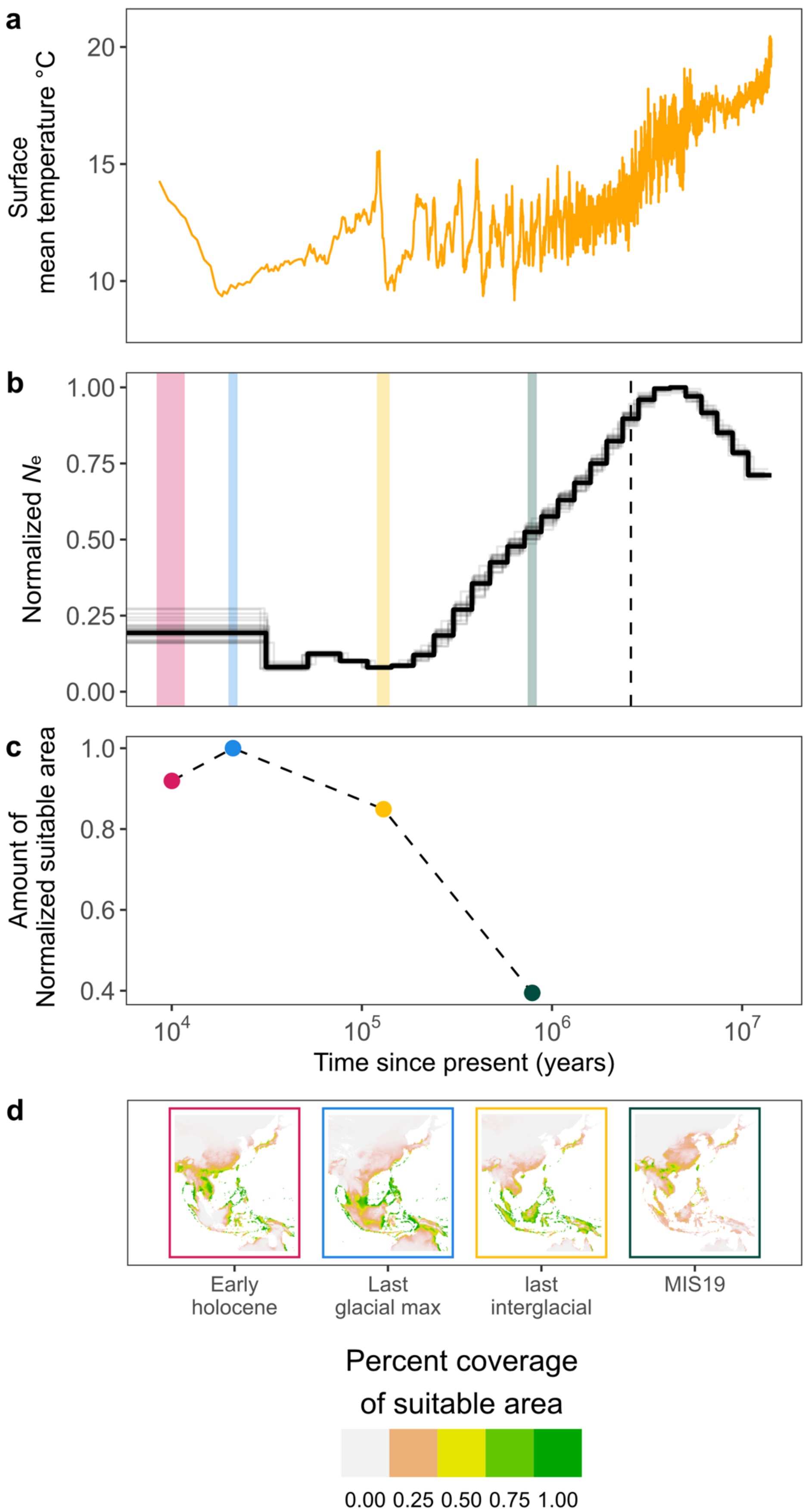

Figure S17. Effective population size trajectories over time and Environmental Niche Modelling for *Pelodiscus sinensis.* Details as in Figure S5.

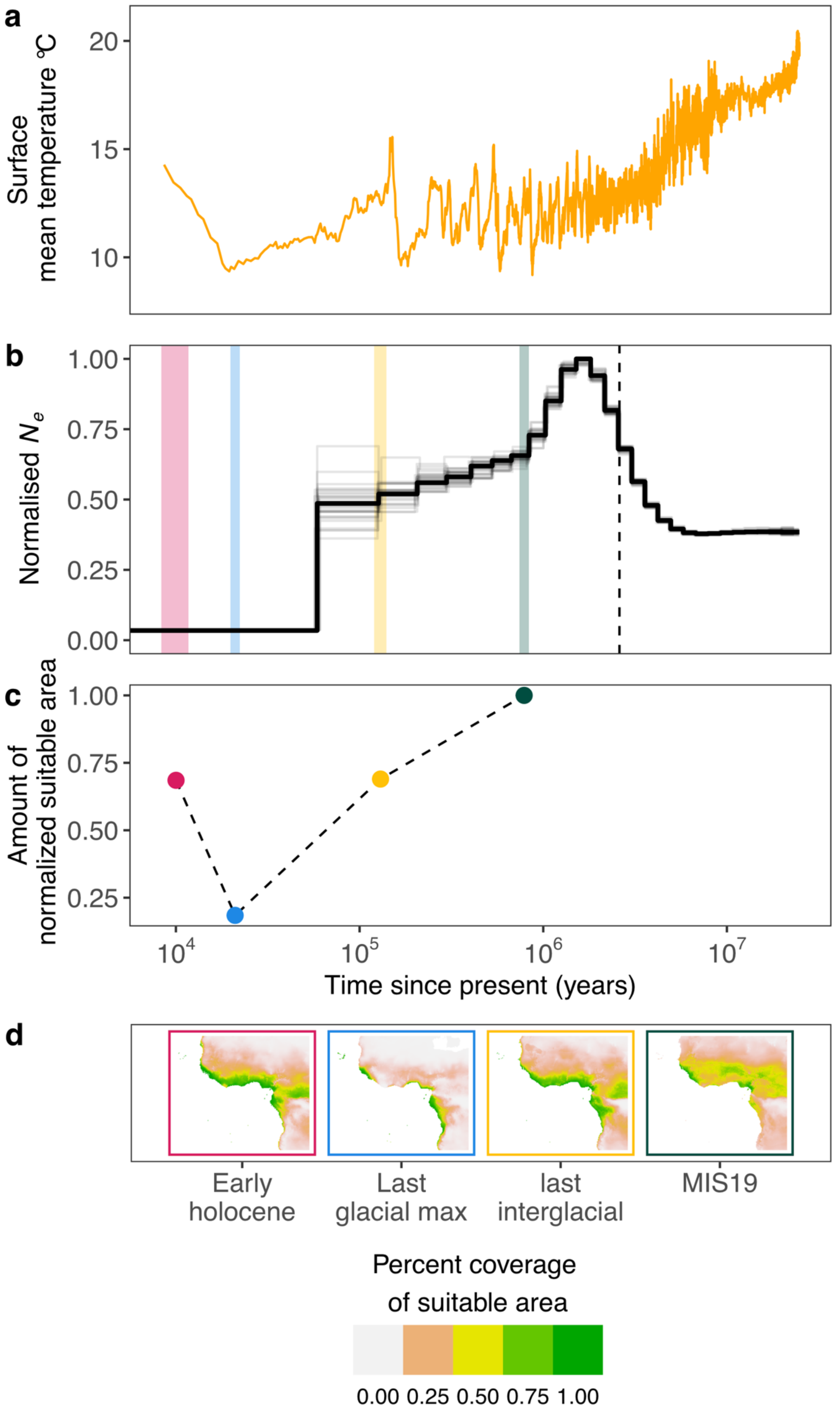

Figure S18. Effective population size trajectories over time and Environmental Niche Modelling for *Pelusios castaneus.* Details as in Figure S5.

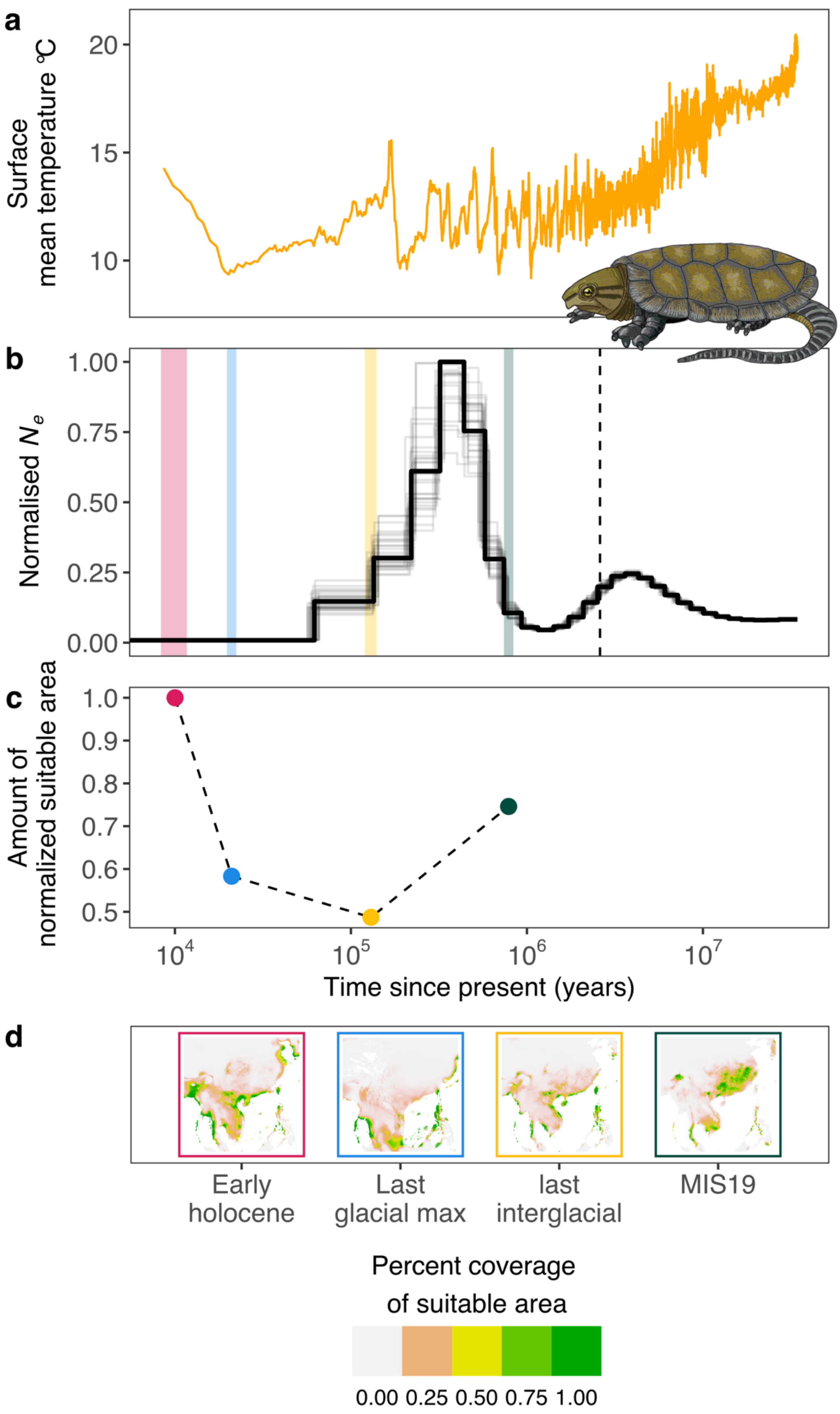

Figure S19. Effective population size trajectories over time and Environmental Niche Modelling for *Platysternon megacephalum.* Details as in Figure S5.

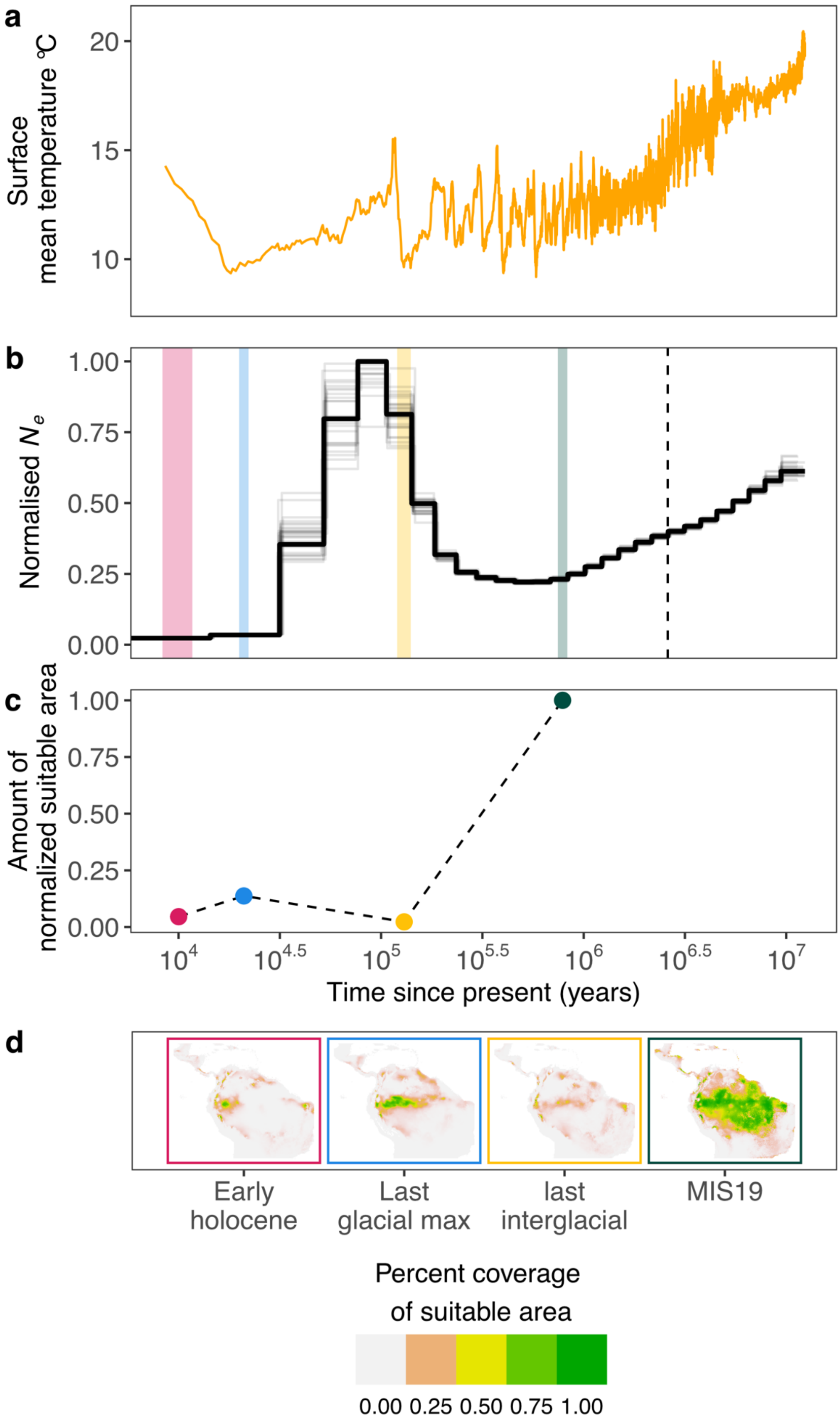

Figure S20. Effective population size trajectories over time and Environmental Niche Modelling for *Podocnemis expansa.* Details as in Figure S5.

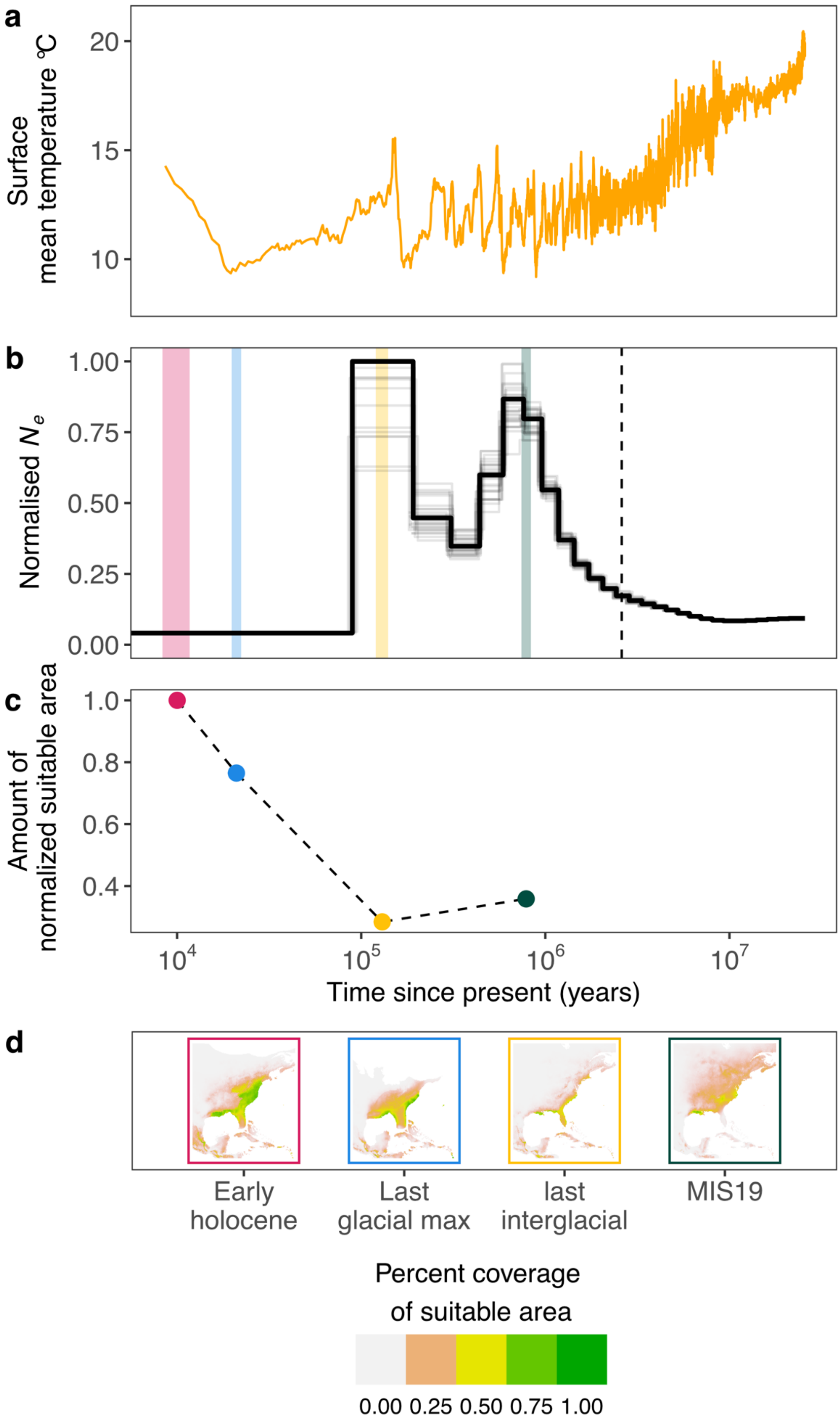

Figure S21. Effective population size trajectories over time and Environmental Niche Modelling for *Terrapene carolina triunguis*. Details as in Figure S5.

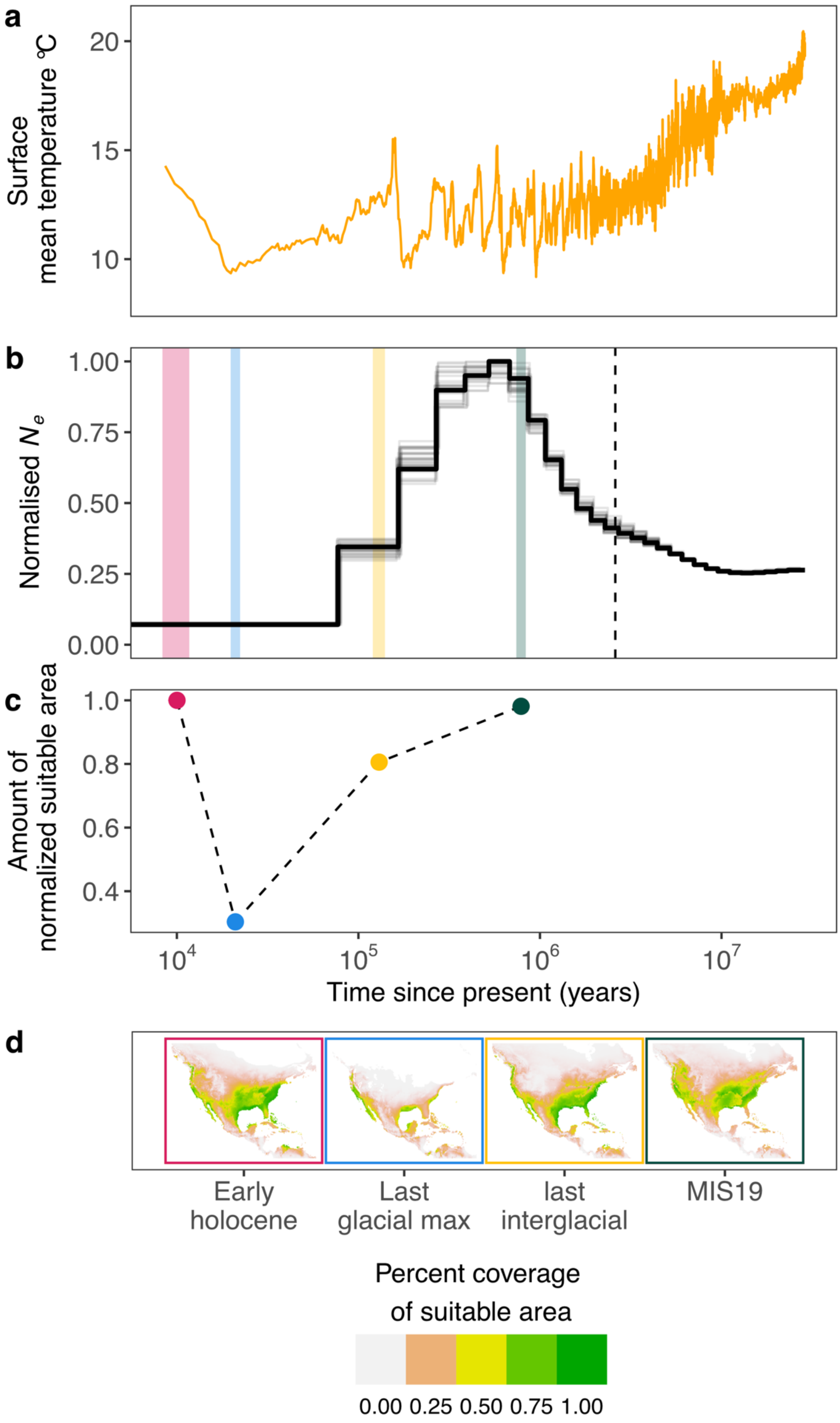

Figure S22. Effective population size trajectories over time and Environmental Niche Modelling for *Trachemys scripta elegans*. Details as in Figure S5.

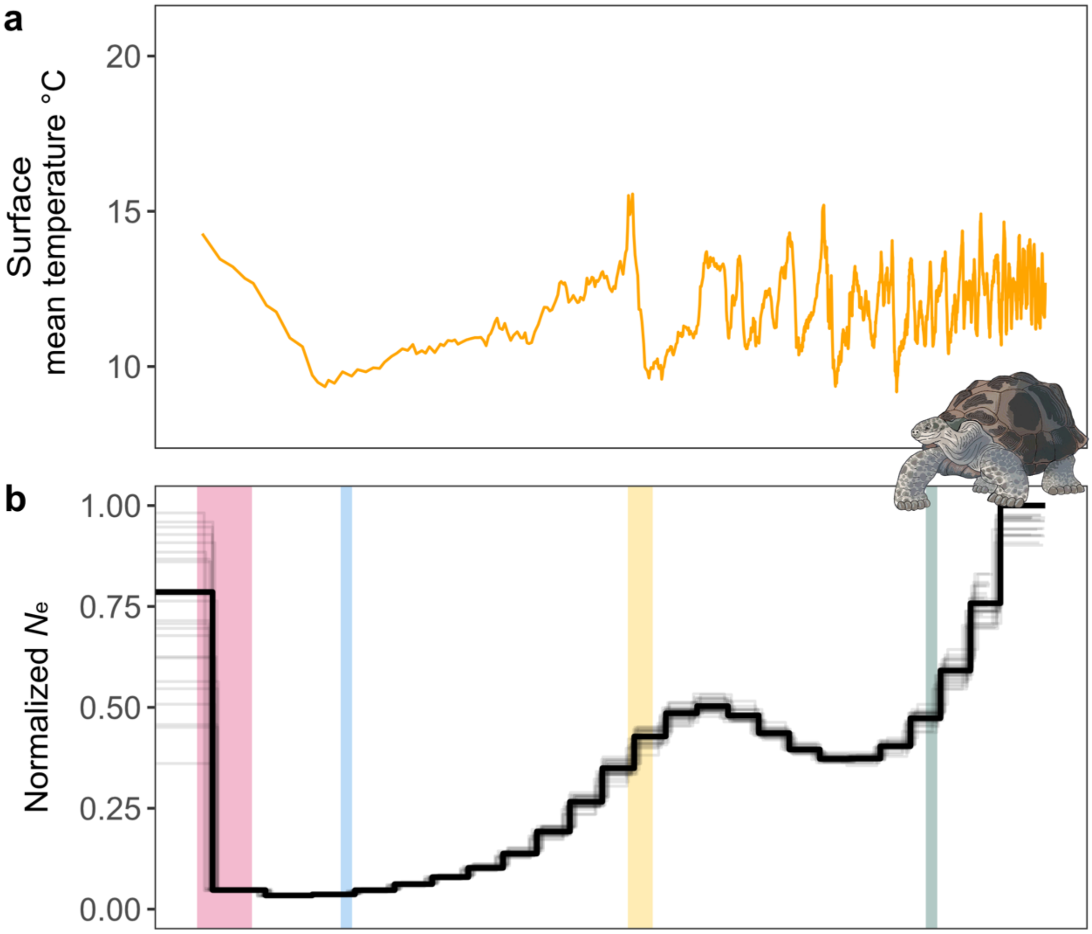

Figure S23. Effective population size trajectories over time in *Chelonoidis abingdonii.* Details as in Figure S5.

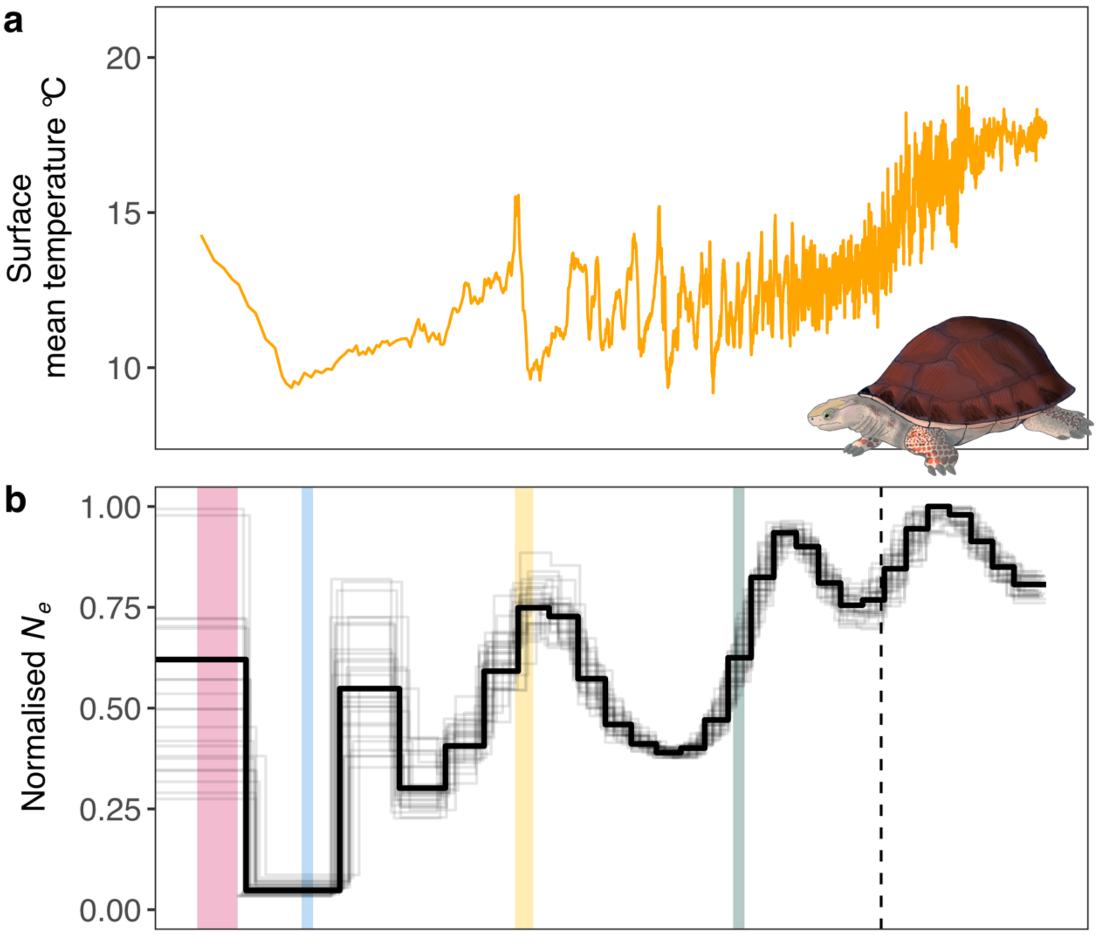

Figure S24. Effective population size trajectories over time in *Cuora mccordi.* Details as in Figure S5.

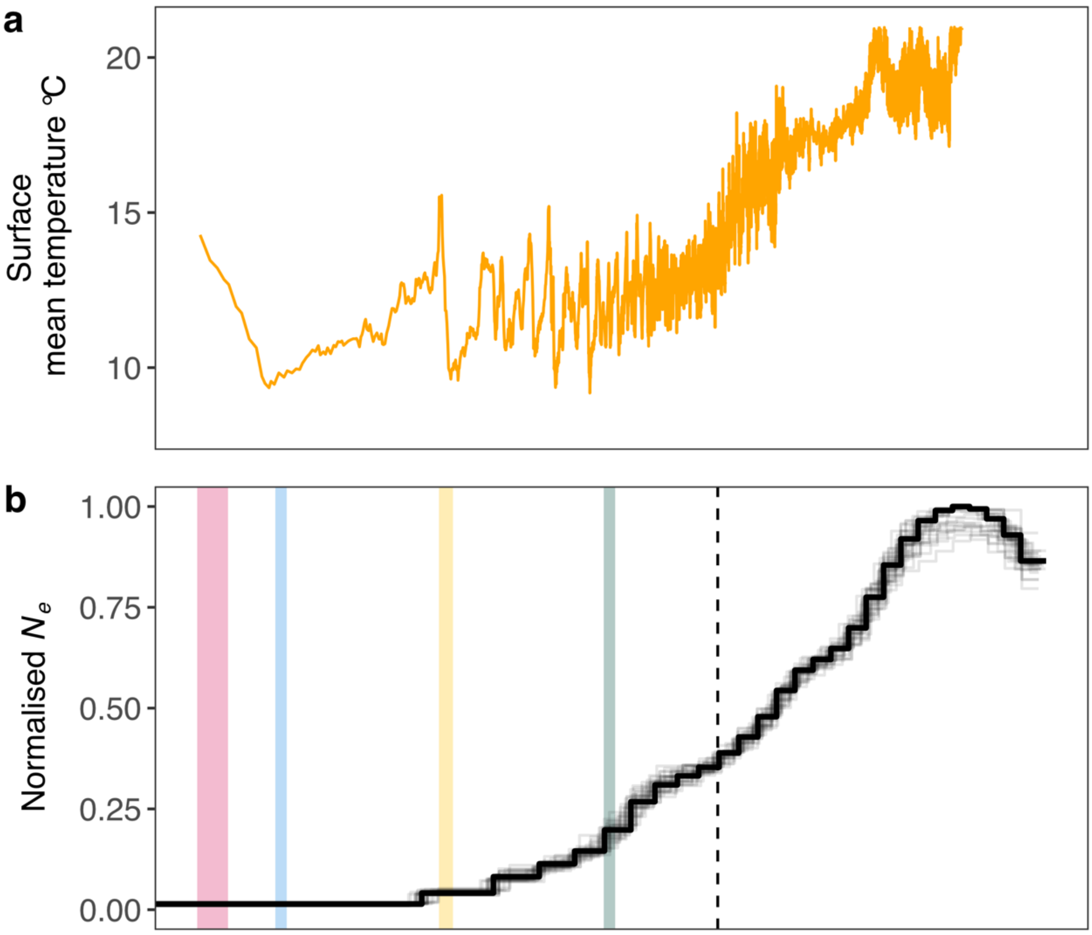

Figure S25. Effective population size trajectories over time in *Rafetus swinhoei.* Details as in Figure S5

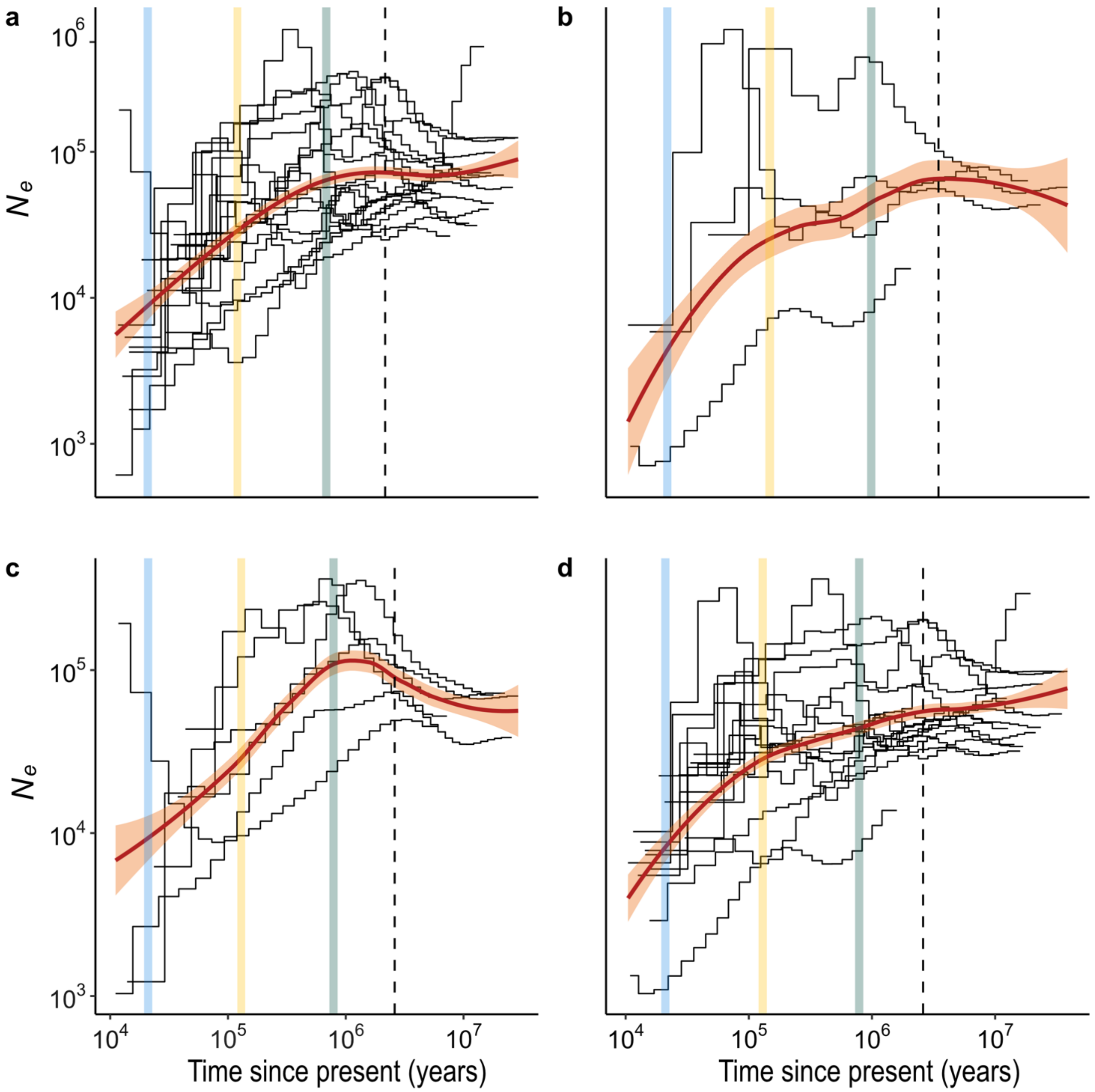

Figure S26. Mean change in N_e from 10 Mya to 10 kya for 22 species of terrapins and tortoises divided in a) freshwater, b) terrestrial, c) temperate and d) tropical species. Each black step line shows changes in N_e trough time for a single species. The red line represents the regression model showing the general pattern of N_e variation through time. The light red-shaded area represents the 95% confidence interval of the linear model prediction. Coloured vertical bars and dashed vertical line as in Figure S5.

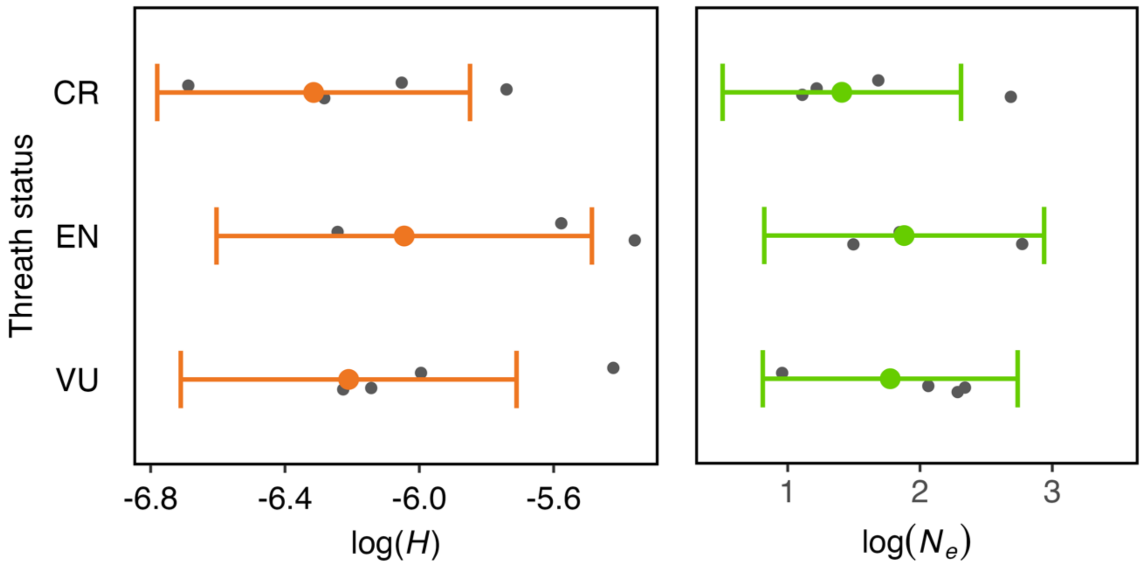

Figure S27: Present suitable area and mean past suitable area estimated using a multiple regression model for Critically Endangered (CR), Endangered (EN) and Vulnerable (VU) species according to IUCN criteria. Orange and green dots and error bars represent model point estimates and 95% confidence intervals, respectively. Raw data points are shown in grey. The Tukey's test found no significant differences among IUCN categories.

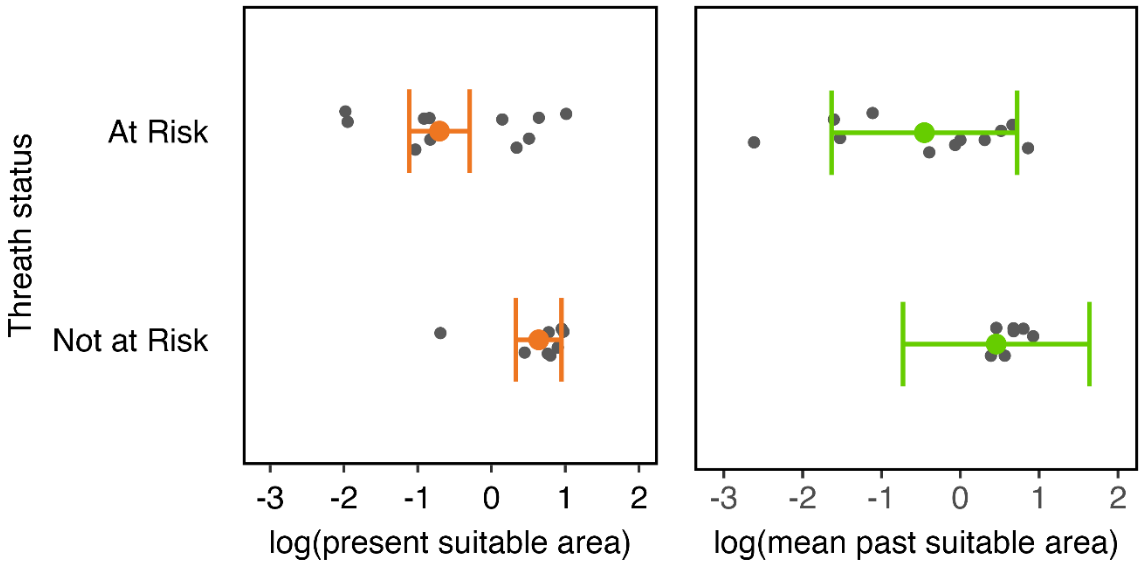

Figure S28: Heterozygosity (*H*) and mean effective population size (N_e) estimated using a multiple regression model for “At risk” (species listed as Vulnerable, Endangered and Critically Endangered according to IUCN criteria) and “Not at risk” (species listed as Least Concern and Near Threatened). Orange and green dots and error bars represent model point estimates and 95% confidence intervals, respectively. Raw data points are shown in grey. The Tukey's test recovered significant differences between at risk and not at risk categories for both present and mean past suitable area.
